# Simulating Honey Bee Large‐Scale Colony Feeding Studies Using the BEEHAVE Model—Part II: Analysis of Overwintering Outcomes

**DOI:** 10.1002/etc.4844

**Published:** 2020-09-22

**Authors:** Farah Abi‐Akar, Amelie Schmolke, Colleen Roy, Nika Galic, Silvia Hinarejos

**Affiliations:** ^1^ Waterborne Environmental Leesburg Virginia USA; ^2^ Syngenta Crop Protection, Greensboro North Carolina USA; ^3^ Sumitomo Chemical, Saint Didier au Mont d'Or France

**Keywords:** BEEHAVE model, Honey bees, Colony overwintering, Large‐scale colony feeding studies, Feeding schedule

## Abstract

Large‐scale colony feeding studies (LSCFSs) aim to assess potential pesticide exposure to and effects on honey bees at the colony level. However, these studies are sometimes affected by high losses of control colonies, indicating that other stressors may impact colonies and confound the analysis of potential pesticide impacts. We assessed the study design and environmental conditions experienced by the untreated control colonies across 7 LSCFSs conducted in North Carolina (USA). Overwintering success differed considerably among the studies, as did their initial colony conditions, amount and timing of sugar feeding, landscape composition, and weather. To assess the effects of these drivers on control colonies' overwintering success, we applied the mechanistic colony model BEEHAVE. Sugar feedings and initial status of the simulated colonies were more important for fall colony condition than were landscape and weather. Colonies that had larger colony sizes and honey stores in the fall were those that began with larger honey stores, were provided more sugar, and had supplemental feedings before the fall. This information can be used to inform the standardization of a study design, which can increase the likelihood of overwintering survival of controls and help ensure that LSCFSs are comparable. Our study demonstrates how a mechanistic model can be used to inform study designs for higher tier effects studies. *Environ Toxicol Chem* 2020;39:2286–2297. © 2020 The Authors. *Environmental Toxicology and Chemistry* published by Wiley Periodicals LLC on behalf of SETAC.

## Graphical abstract

Data from 7 large‐scale colony feeding studies for honey bees demonstrated that colonies with more fall honey (and other factors) were more likely to survive the winter. Using a calibrated BEEHAVE colony model, we ran scenarios suggesting that fall colony condition was most impacted by the amount and date of feedings, as well as the initial conditions of hives.

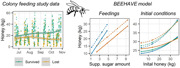

## INTRODUCTION

The honey bee large‐scale colony feeding study (LSCFS) is a novel higher tier study design for the determination of potential effects of pesticides on free‐foraging whole colonies during and after dietary intake of a known pesticide concentration (e.g., Overmyer et al. 2018; Thompson et al. 2019). The LSCFSs include the assessment of overwintering success (i.e., survival and spring growth) of colonies following the exposure period. In recent risk assessments, regulatory authorities in North America have used LSCFS data to quantify effects on honey bee colonies from chronic exposure of neonicotinoid insecticides (US Environmental Protection Agency 2016, 2017) and other active ingredients. Despite their continued use in regulatory risk assessments, no formal regulatory protocol exists for conducting the LSCFSs.

A major problem in some LSCFSs is low overwintering success in untreated control colonies. This complicates the interpretation of effects to the treated colonies (US Environmental Protection Agency 2017), suggesting that factors other than pesticides play an important role in driving overwintering colony success. Honey bee colony health and overwintering success have been linked to various external factors such as beekeeping management practices, weather, and bee resource availability in the landscape (e.g., Genersch et al. 2010; Döke et al. 2015; Switanek et al. 2017; Beyer et al. 2018; Kuchling et al. 2018; Honey Bee Health Coalition 2019). Honey stores and adult bee numbers are generally recognized and reported as important measures of colony health, especially before overwintering (e.g., Genersch et al. 2010; Austin 2014; Döke et al. 2015). This is consistent with the science‐based recommendations for best beekeeping management practices, suggesting that, among other efforts, attempts to reduce overwintering losses should focus on enhancing colony strength (i.e., numbers of adult bees and developing eggs, larvae, and pupae, collectively referred to as brood) and food stores (i.e., nectar, honey, and pollen) in the fall (Steinhauer et al. 2014).

In the present study, we addressed the problem of understanding LSCFS control colony overwintering losses first by analyzing for associations within data from control colonies in 7 LSCFSs. All studies were conducted in North Carolina (USA) between 2013 and 2017. The data consisted of measurements from colony condition assessments (CCAs), in which visual frame coverage was used to estimate numbers of bees in different stages as well as honey and pollen cells. The CCAs were conducted at prescribed intervals between May (after initial setup of the colonies) and October in the first year of each study, and in March and/or April of the following year to assess overwintering success. Across studies, we compared the different aspects of the LSCFSs with their overwintering outcomes, as well as with their colony conditions at the end of the first year.

Multiple factors differed among LSCFSs, not allowing for the identification of clear associations between potential causes and effects from the available data alone. In modeling approaches, all factors of a simulated system can be fully controlled, allowing for analysis of the impacts of isolated factors and their interactions with a system over time. Multiple honey bee colony models have been introduced with the aim of improving our understanding of the interplay of many processes and factors in this complex system (Becher et al. 2013, 2014; Khoury et al. 2013; Betti et al. 2017; Kuan et al. 2018). In the present study, we used the mechanistic honey bee colony model BEEHAVE (Becher et al. 2014) to systematically assess different factors impacting control colony conditions in LSCFSs.

The BEEHAVE model has explicit representations of foraging in the landscape and in‐hive processes such as brood raising (i.e., development of eggs, larvae, and pupae; Becher et al. 2014). Environmental conditions and beekeeping activities can be explicitly represented in the model, which was previously calibrated and validated with data from the control colonies of the 7 LSCFSs (see details in Schmolke et al. 2020, this issue). Data from these colonies were grouped and analyzed over time, showing that the last assessment in the fall prior to overwintering was the most predictive of overwintering outcome. During this first year of studies prior to overwintering, the calibrated BEEHAVE model showed good model performance with respect to estimating adult worker bee numbers and honey stores in the colonies. The validation of the BEEHAVE model emphasizes that the mechanistic representation of colony processes in the model are successfully capturing colony dynamics in the first year of the LSCFSs. The calibrated BEEHAVE model was used to simulate colony dynamics in response to varying environmental conditions and beekeeping activities reported in the LSCFSs. Although we are using a calibrated and validated BEEHAVE model to run these simulations, these analyses are not fully dependent on how well the simulations represented LSCFS data, because the focus is on the differences in colony conditions resulting from varying factors.

The goal of conducting simulations with BEEHAVE was to identify the most important factors impacting control colony condition within LSCFSs in the fall, and in turn, to inform study design aspects that increase the likelihood of overwintering success in control colonies. We simulated the range of landscape compositions, weather conditions, initial control colony conditions, and feeding patterns reported in the 7 studies. In 2 sets of simulations, we varied multiple factors and analyzed their impact on colony conditions in the fall, prior to overwintering.

## MATERIALS AND METHODS

### Analysis of data from LSCFSs

The LSCFSs are designed to assess the impacts of pesticide exposure on honey bee colonies over a full cycle of a foraging season and subsequent overwintering period. The LSCFS design involves a relatively large number of replicates (e.g., 12 apiary locations, each with one colony/treatment), treatment levels (5 treatments + controls), and colony condition assessments (e.g., 8–10 assessments over approximately 12 mo, including overwintering). In the evaluated LSCFSs, the pesticide exposure was controlled by feeding pesticide‐spiked sugar syrup to the colonies over a 6‐wk period during the summer, whereas control colonies were fed only sugar syrup. Supplemental sugar was provided to the colonies between August and April as standard practice to help achieve sufficient honey stores for winter survival. A more detailed description of the LSCFSs used for the present study can be found in Schmolke et al. (2020; this issue).

Data from the 7 LSCFSs were used to examine factors associated with control colony overwintering survival or loss. Prior to winter, signs may be discernable that could identify colonies more at risk. The time period of interest in our analysis is therefore the first year of each study, between colony placement in study apiaries (late June or early July) and the end of the honey bee foraging season (late October).

Several CCAs were conducted during the first year of each study (Supplemental Data, Figure S1). Measurements from all LSCFSs' control colonies were graphed together to more robustly identify dates at which different metrics may be predictive of overwintering outcome. Colonies that died prior to October were omitted. The time lines began from each study's first CCA conducted after placement of the colonies in the apiary and ended with the last CCA prior to overwintering.

Two locally estimated scatterplot smoothing (LOESS) curves were fit to these points: one to colonies that survived the winter, and one to those that did not. These curves smoothed the points to aid in visually distinguishing any variability between the 2 outcomes at different times across the season. This was repeated for honey stores (in kg, where 1 kg ≈ 2000 cells), adult bees, pupae, and the ratio of adult bees to honey stores. Pollen, larvae, and egg measures were not used due to high measurement error (see Schmolke et al. 2020, this issue). Data on *Varroa* *destructor* mite and *Nosema* spp. infestations were not used due to sparse data availability (only estimated at 2–3 time points/study during the foraging period). Graphing was performed in R software with the package ggplot2 (Wickham 2016; R Core Development Team 2019; LOESS based on weighted least squares at span = 0.75 with 95% confidence interval based on Cleveland et al. 1992).

Based on these time lines, all colonies' final CCAs prior to winter were further examined to determine whether fall colony condition could serve as a predictor of overwintering outcome. The fall CCAs occurred between 15 and 28 October across the 7 LSCFSs (Supplemental Data, Figure S1). These end‐of‐season measurements from the 4 colony condition metrics (honey stores, adult bees, pupae, and the ratio of adult bees to honey stores) were graphed, colored by overwintering success. Thresholds identifying likely overwintering losses were defined at levels below which approximately 90% of colonies died. Similarly, thresholds for survival above which approximately 90% of colonies survived were identified. Using these thresholds as predictions, outcomes were statistically tested for difference from 50% using Fisher's exact tests and exact binomial tests (R Core Development Team 2019).

This step suggested that the number of adult bees and size of honey stores in October were partially predictive of overwintering outcome. Therefore, if a study aspect was found to be significantly explanatory of fall adult bees and/or honey, it could in turn be explanatory of overwintering success. Thus fall colony condition was the focus of the subsequent modeling analyses.

### Factors impacting fall colony conditions in initial BEEHAVE simulations

Although overwintering outcomes varied among the colonies in these 7 LSCFSs, known variability in several aspects of the LSCFSs and the apiaries within them prevented a clear and targeted comparison of specific potential causes of different loss rates. The studies varied by year, by specific location, and in the design aspects of feeding schedules and grouping based on initial colony sizes (see Supplemental Data, Figure S2 for a summary). Often, these characteristics were tied together, or confounded. With unique aspects co‐occurring, it was not possible to know which aspect, or what combination, affected fall colony results. Variability existed within each study as well as among studies.

In an initial set of simulations, we used the mechanistic honey bee colony model BEEHAVE to isolate the potential impact these different characteristics had on fall control colony condition. The ranges of 4 of these aspects of colony variability were tested one by one systematically with BEEHAVE as follows: 1) colony conditions observed at the time of study initiation, including initial number of adult worker bees, brood, and honey and pollen stores per hive; 2) feeding timing and amount, including both treatment feedings and supplemental feedings with sugar syrup; 3) landscape composition around apiaries, reflecting spatial and temporal bee resource availabilities; and 4) weather impacting daily foraging hours available to simulated foraging bees. Each is represented by a BEEHAVE input file unique to each scenario (discussed in Schmolke et al. 2020, this issue). In this initial set of BEEHAVE simulations, all 4 aspects were examined for potential impact on fall colony conditions using the calibrated BEEHAVE model (Schmolke et al. 2020, this issue).

For this set of initial simulations, different inputs within each of these 4 aspects were identified to represent the ranges of variability across LSCFSs. The combinations of all these ranges and aspects were run in BEEHAVE (see list and details in Supplemental Data, Section 2.0). All combinations' resulting counts of adult bees and amounts of honey in the fall were graphed by variable to inform whether the different levels of each led to more or fewer adult bees and/or honey stores on 21 October. This date represents the approximate mean date of the final fall colony condition assessments among all LSCFSs. Based on the ranges of variability observed in these graphs, feeding schedules and initial conditions were the 2 aspects targeted for more in‐depth simulations.

### BEEHAVE simulations targeting study design options

Because feeding schedules and initial conditions were identified as drivers impacting fall colony conditions in the initial simulations, their characteristics were broken down, and then systematically varied in this set of targeted BEEHAVE simulations. This allowed each characteristic of each factor to be isolated and tested for potential effects on fall colony condition, akin to a sensitivity analysis. Combining all characteristics also informed their relative impact on fall colony conditions, and any interactions among them.

Feeding schedules varied across the 7 studies in terms of timing and amount of sugar fed. There were 2 periods of feeding: treatment feeding early in the season, and supplemental feeding later in the season. Although the treatment feeding period had a separate original goal within these studies of exposing some colonies to a pesticide, our analysis only used data from the untreated control colonies, which were fed a sugar solution at the same schedule and amount as the others. Bees in control colonies consumed the great majority of this untreated syrup provided, which suggests that the colonies' sugar needs were not fully met by the treatment feedings, and additional nectar was collected in the landscape. For the targeted BEEHAVE simulations, 2 treatment feeding amounts of 0.82 and 1.64 kg honey equivalent were used (respectively corresponding to 1 or 2 L of solution comprising 1 part sugar, 1 part water by weight). To standardize the timing, 28 June was chosen as the date for study initiation in all simulations, because it was the mean first assessment date across studies (see Schmolke et al. 2020, this issue). The average amount of time before the first treatment feeding in the studies was 6 d, so it was simulated on 4 July. All simulations had 12 treatment feedings thereafter, 3 to 4 d apart.

Supplemental feeding began later in the year and differed by start dates, frequencies, durations, and amounts of sugar fed across studies. These details are visualized over time in the Supplemental Data, Figure S3. The amount of sugar syrup consumed by each colony was reported in one LSCFS, which showed that most colonies consumed all the sugar syrup, with variability by colony and date. The range of supplemental feeding start dates was represented in these simulations by 2 standardized dates, 23 August and 23 September. Although in 3 studies, supplemental feedings began after 21 October, the fall date of focus in our analysis, this later scenario was not simulated because it led to consistently worse fall colony conditions in an earlier set of simulations (see Supplemental Data, Section 3.2). All supplemental feedings were applied on the 8th and 23rd of each month, ending on 8 October. Thus, in the hypothetical scenarios beginning on 23 August, bees were fed 4 times, and in those beginning on 23 September, bees were fed twice in the BEEHAVE model. For the amount of sugar provided/feeding, 8 different combinations were chosen for the simulations (Table 1). These reflect 4 different total amounts of sugar fed starting on 2 different dates, to allow for a comparison between start dates while keeping the total sugar amount constant.

**Table 1:** The 8 supplemental feeding combinations applied in the targeted BEEHAVE simulations

Sugar/supplemental feeding (kg honey equivalent)	Start date	No. of feedings	Total sugar fed (kg honey equivalent)
1.18	23 August	4	4.7
2.35	23 September	2	4.7
2.35	23 August	4	9.4
4.7	23 September	2	9.4
3.52	23 August	4	14.1
7.05	23 September	2	14.1
4.7	23 August	4	18.8
9.4	23 September	2	18.8

Once honey bee colonies had been placed in their study apiaries, the first CCA was conducted between 20 June and 6 July of the study year, recording each colony's number of adults, pupae, larvae, eggs, pollen cells, and honey cells. The values from all colonies across all studies were first combined into distributions, one/metric. The 5th, 50th, and 95th percentiles of initial honey stores and initial numbers of adult bees were used as inputs in these simulations. The initial measures of pollen, pupae, and uncapped brood (larvae + eggs) were omitted due to their considerably smaller effects, as found in an earlier analysis (see Supplemental Data, Section 4.0 and Figure S24); pollen, larvae, and eggs also had high measurement error (see Schmolke et al. 2020, this issue). Instead, these metrics were assigned their median values in all scenarios. The combination of the 95th percentile of adults and the 5th percentile of honey was omitted because it was not observed in any study colonies.

In all, the 2 treatment feeding amounts, 8 supplemental feeding schedules, and 8 initial conditions combined to create 128 hypothetical scenarios. Each was applied to 8 apiaries, which were chosen to span studies, and to vary by fall condition and overwintering success of the control colonies (Supplemental Data, Table S1). The other unique aspects of these apiaries that varied in BEEHAVE—their weather and surrounding landscape—were used as‐is, such that the only modification was their assigned hypothetical scenario. Applying the same set of feeding scenarios to multiple apiaries allows for evaluation of the consistency of response to aspects applied in different circumstances, and consideration of the remaining unexplained variability.

Each of these was then repeated 20 times to incorporate stochasticity, totaling 20 480 BEEHAVE runs. The numbers of adult bees and the sizes of honey stores on 21 October were pulled from each of the run outputs, and the means of each set of 20 replicates were calculated, representing fall colony condition. These 1024 values served as the basis of the statistical analysis.

### Statistical analysis of BEEHAVE outputs targeting study design options

The goal of our statistical analysis was to identify whether the different feeding schedules and initial colony conditions affected the resulting numbers of adult bees and honey stores in the fall, and if so, to quantify those effects. The scenarios that produced surviving colonies in the fall in all 20 replicates (>0 adult bees, 92% of scenarios) were analyzed separately from the scenarios that produced some or all replicates with dead colonies (0 adult bees, 8% of scenarios) prior to 21 October, due to substantial differences in resulting values.

The main analysis used data from the scenarios that produced surviving fall colonies in all replicates. Two linear mixed‐effects (LME) models were built, which combined least‐squares multiple regression and analysis of variance to account for a grouping structure in the data. The 8 apiaries, each with known inherent uniqueness, were the random‐effect groups statistically represented by intercept shifts. The dependent, or predicted, variable in the first model was the number of adult bees on 21 October simulated with BEEHAVE, and the predicted variable in the second model was the amount of honey in colonies on 21 October in BEEHAVE.

The following independent (fixed) variables and interactions were tested in each LME model: the initial number of adult bees and kg of honey in the colony, the amount of sugar/feeding (as kg of honey equivalent) during treatment and supplemental periods, the start date of supplemental feedings (as number of days after 23 August, the first date applied), and the total amount of sugar in supplemental feedings. Analyses were performed in R software (R Core Development Team 2019) with the package lmerTest (Kuznetsova et al. 2017). Residual plots were checked for linearity, homoscedasticity, and normality. A measure of goodness of fit for LME models was calculated and reported (*R* ^2^_β_, Kenward–Roger approach; Edwards et al. 2008; Jaeger 2017), and trends were graphed.

In addition, the hypothetical combined scenarios that produced at least one replicate outcome with 0 fall adults were examined in a separate binary analysis. These scenarios were systematically binned by apiaries as well as by feeding and initial condition characteristics, to identify levels and combinations with the highest risk of producing fall losses.

## RESULTS

### Analysis of data from LSCFSs

**Figure 1:**
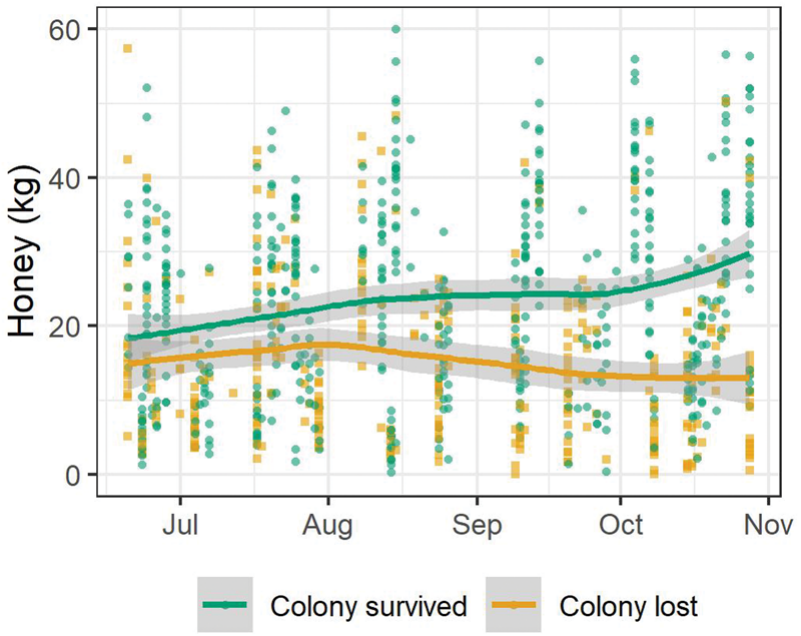
Time line of honey stores from all colonies as measured during all condition assessments in the first year of each study, graphed by shared month and day. The lines are corresponding locally estimated scatterplot smoothing curves, with 95% confidence intervals as gray shaded areas. Honey stores in surviving colonies are the most different from lost colonies at the latest date.

When all LSCFSs' colony metrics were graphed over time, the colonies that ultimately survived the winter were partially distinguishable from those that did not. Honey stores of colonies that survived were often larger than those of colonies that died, with the greatest difference visible in the latest part of the season (Figure 1). Honey stores demonstrated the widest and most consistent difference between groups, but the remaining measures also showed some distinction (Supplemental Data, Figure S4). The number of adult bees was similar between overwintering outcome groups until the end of the season, when surviving colonies had relatively more adult bees. Surviving colonies also had a greater ratio of honey/adult bee, as well as somewhat more pupae at the end of the season. These colony metrics' end‐of‐season values were therefore the basis for further analysis.

**Figure 2:**
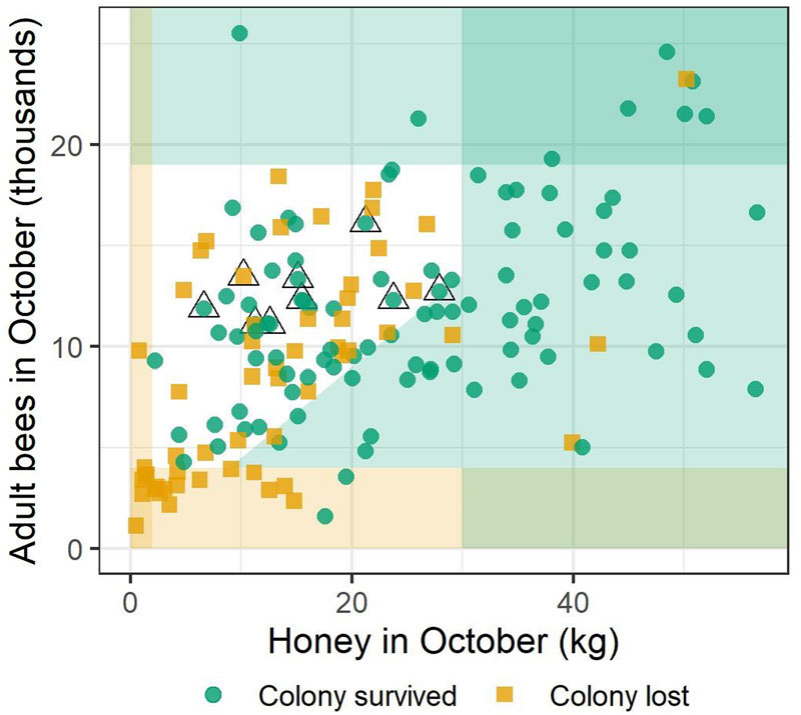
All colonies from all large‐scale colony feeding studies plotted by the number of adult bees and size of honey stores in the fall (October 15–28). Orange shading delineates areas below loss thresholds, and green shading delineates areas above survival thresholds. Black triangles mark colonies surpassing the pupae survival threshold.

Using these end‐of‐season values, thresholds indicative of likely overwintering loss or success were identified. All 156 colonies' fall values were grouped by loss (56) or survival (100; Supplemental Data, Figure S5) and plotted in Figure 2 by their fall colony and honey store sizes. All colonies with <2 kg of fall honey died over the winter (7/7 colonies), serving as a loss threshold. Complementarily, 92% of colonies with >30 kg of honey survived the winter, serving as a survival threshold. This threshold provided a prediction for the most colonies (39). The Supplemental Data, Table S2 lists these counts in a contingency table, from which a Fisher's exact test was run. Together, these honey thresholds were statistically significant in predicting overwintering outcome at an average of 93% accuracy (*p* < 0.0001).

Using the number of adult bees as an indicator for overwintering success, 90% of colonies with <4000 adult bees in the fall died over the winter, whereas 89% of colonies with >19 000 bees survived. These loss and survival thresholds for adult bees provided a prediction for 29 colonies in total, and were 90% accurate (Supplemental Data, Table S2; Fisher's exact test *p* < 0.0001). Both the honey and adult thresholds combined provided a prediction for 40% of the colonies from the LSCFSs (63/156). In Figure 2, these loss thresholds are delineated by the shaded orange areas, and success thresholds by the green horizontal and vertical areas.

The ratio of grams of honey/adult bee provided a prediction for an additional 10% of the colonies. A high ratio was predictive of success among the remaining colonies. When each bee had at least 2.22 g of honey on average for overwintering (equivalent to 450 bees sharing at least 1 kg honey), 88% of colonies survived (Supplemental Data, Table S2; exact binomial test *p* = 0.004). The green shaded triangle in Figure 2 depicts this success threshold. A ratio was not predictive of losses due to similar frequencies of survival and loss at low values.

Finally, among the colonies unexplained by the honey, adult bee, or ratio thresholds, 89% of colonies with at least 11 000 pupae in the fall survived the winter. This pupae survival threshold assigned a prediction to an additional 6% of the colonies (Supplemental Data, Table S2; exact binomial test *p* = 0.04), depicted by black triangles in Figure 2. Few pupae were not predictive of overwintering deaths.

Using all thresholds, 56% of all colonies were assigned a prediction of overwintering outcome that was accurate 91% of time. The remaining 68 points in the white area of Figure 2 could not be assigned a prediction with these data; they were mixed in outcome such that 44% died over the winter.

### Factors impacting fall colony conditions in initial BEEHAVE simulations

**Figure 3:**
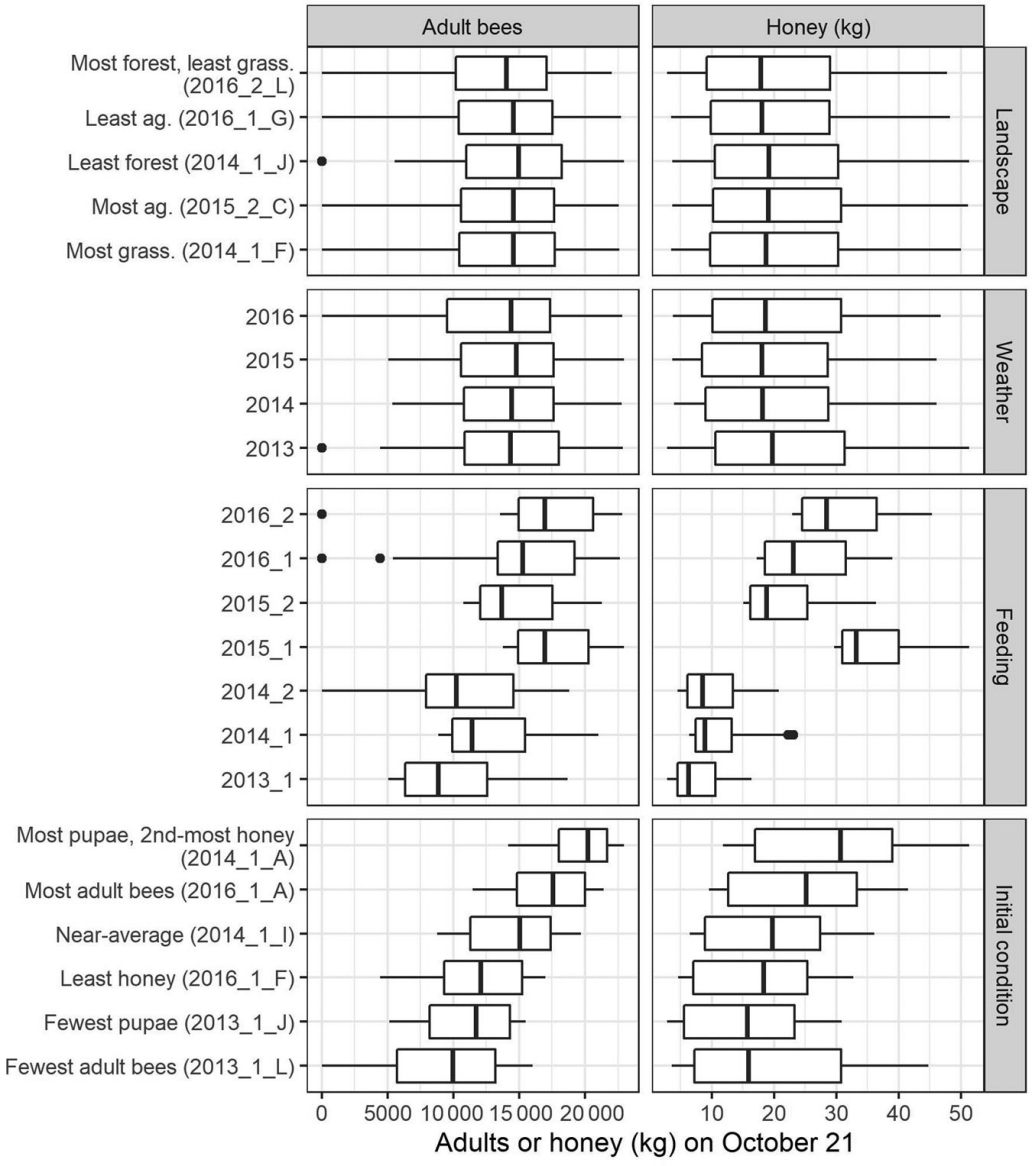
Box plots of resulting fall adult bees (left column) and honey stores (right column) from the initial set of simulations, separated by different aspects as labeled along the right. The *y*‐axis labels can include study and apiary labels ([beginning year]_[study number]_[apiary ID]). Each plot is inclusive of all results. Boxes extend from the 25th to 75th percentile (interquartile range [IQR]), with the central line at the median. Whiskers extend up to 1.5 × the IQR, with points beyond.

The results of the initial set of simulations are graphed by variable in Figure 3. Overall, the different landscape compositions and weather years produced negligibly different ranges of fall adult bees or honey stores, as is visible in the large overlap across boxes in the top 2 rows of plots in Figure 3. In contrast, the ranges of resulting fall adult bees and honey stores shifted among different studies' feeding schedules and initial conditions. The feeding schedules of the studies in 2013 and 2014 produced the fewest fall adult bees and least honey on average, whereas those of studies 2015_1 and 2016_2 had the most. The initial conditions showed that the colonies beginning with fewer adult bees, pupae, or honey also had low resulting fall measures, and vice versa, with the near‐average starting condition between the 2 extremes. Note that more fall honey can indicate a productive colony, or a colony with bee mortality in which honey stores remain unconsumed.

Because feeding schedules and initial conditions notably impacted fall colony health metrics, their characteristics were then simulated in more detail.

### Statistical analysis of BEEHAVE outputs targeting study design options

Differences in fall conditions emerged as a result of systematically varying the study design characteristics targeted in this set of BEEHAVE simulations. Among all hypothetical scenarios run in the set of targeted simulations, 8% (80 scenarios of 1024) resulted in replicates that died before 21 October. These all shared the commonality of having the low 5th percentile amount of initial honey, suggesting that colonies beginning with too little honey may not survive to the fall (see more details in Supplemental Data, Table S3). In the analysis in this section, we present simulation results from the remaining 92% of scenarios in which all replicates had surviving bees in the fall.

First, the LME model explaining the number of adult bees in the fall (Table 2) suggested that most variability across hypothetical combined feeding and initial condition scenarios was explained (*R* ^2^_β_ = 98%) by a combination of timing and sugar amount during both treatment and supplemental feedings, as well as the initial amount of honey and number of adult bees. There were also interactions between supplemental feeding timing and sugar amount, as well as between initial honey and initial adult bees. An additional interaction of initial honey amount and treatment feeding amount suggested that when more treatment‐feeding sugar was provided to colonies with more initial honey stores, there were slightly fewer fall adult bees, which is further explained in the *Discussion* section. Residuals appeared homoscedastic and normally distributed (residual plot in Supplemental Data, Figure S6).

**Table 2:** Results of the linear mixed‐effects model explaining variability in the number of adult bees on October 21 (B), as output by BEEHAVE from the targeted simulations^a^

Random effects: Group	Standard deviation		
Apiary (8 total)	690		
Residual	481		

Fixed effects: Variable	Coefficient	Standard error	*p* value
Intercept	4682.7	272.757	3.3e‐09
Initial number of adult bees in colony, in thousands (IB)	100.32	4.544	<2e‐16
Initial amount of honey store in colony, in kg (IH)	234.13	4.443	<2e‐16
Treatment feeding amount of sugar per feeding, in kg honey equivalent (TA)	3793.6	72.125	<2e‐16
Supplemental feeding first date, with August 23 as day 0 and each additional day + 1 (SD)	9.3690	2.475	1.6e‐04
Supplemental feeding amount of sugar fed, in kg honey equivalent (SA)	1061.47	16.859	<2e‐16
IB × IH	–2.3496	0.175	<2e‐16
SD × SA	–24.378	0.608	<2e‐16
IH × TA	–14.349	2.905	9.3e‐07

^a^ Equation form: B = Intercept + IB + IH + TA + SD + SA + (IB** **×** **IH) + (SD** **×** **SA) + (IH** **×** **TA).

Because these statistical analyses are based on data from a set of simulations, the *p* values are not the focus and are not representative of chance. The number of model repetitions too strongly dictates significance. In addition, we know that changing input parameters changes output values, so the null hypothesis of similarity is already known to be false (White et al. 2014). Instead, for the present study, we focused on the coefficients to quantify the average impact of each variable on adult bees or honey stores on 21 October, controlling for all other variables. The random effects' standard deviations reflect the remaining unexplained variability after controlling for the fixed‐effect variables.

Second, the LME model explaining kg of honey in a colony in the fall is shown in Table 3. Together, all factors produced a high level of explanation (*R* ^2^_β_ = 99%). This honey LME model has the same variables and structure as the adult bee LME model above, except that the initial honey value is squared. This transformation was necessary to reflect the nonlinear jump in fall honey levels observed at the 95th percentile initial honey and the higher treatment feeding level. Residuals appear homoscedastic and normally distributed (Supplemental Data, Figures S7 and S8). As with the previous adult bee LME model, the *p* values are not representative of chance because they are based on simulations; instead, the focus is on the coefficients to quantify average impacts.

**Table 3:** Results of the linear mixed‐effects model explaining variability in the size of honey stores on October 21 (H), as output by BEEHAVE from the targeted simulations^a^

Random effects: Group	Standard deviation		
Apiary (8 total)	0.636		
Residual	0.8334		

Fixed effects: Variable	Coefficient	Standard error	*p* value
Intercept	–1.570	0.283	3.5e‐05
Initial number of adult bees in colony, in thousands (IB)	0.1504	0.00572	<2e‐16
Initial amount of honey store in colony, squared, in kg^2^ (IH^2^)	0.003350	0.000173	<2e‐16
Treatment feeding amount of sugar per feeding, in kg honey equivalent (TA)	3.949	0.0962	0.0063
Supplemental feeding first date, with August 23 as day 0 and each additional day + 1 (SD)	0.01176	0.004289	<2e‐16
Supplemental feeding amount of sugar fed, in kg honey equivalent (SA)	4.378	0.0292	<2e‐16
IB × IH^2^	–0.0001521	6.48E‐06	<2e‐16
SD × SA	–0.06092	0.00105	<2e‐16
IH^2^ × TA	0.005860	0.000113	<2e‐16

^a^ Equation form: H = Intercept + IB + IH^2^ + TA + SD + SA + (IB × IH^2^) + (SD × SA) + (IH^2^ × TA).

**Figure 4:**
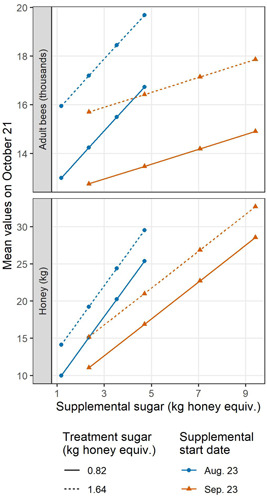
Mean effects of feeding schedules on fall colony metrics from the linear mixed‐effects models, based on the targeted set of BEEHAVE model simulations. These values are set at the median levels of initial adult bees and initial honey stores, as an example.

Next, both LME equations were used to quantify and interpret mean increases/decreases in adult bee and honey amounts on 21 October. The values are not intended to serve as precise predictions, but to provide relative comparisons of impact among variables. The mean effects of the different feeding characteristics are graphed in Figure 4. Values are shown at median levels of initial adult bees and initial honey stores, as an example. To see all results, see the Supplemental Data, Section 5.0.

During supplemental feeding, providing more sugar led to more adults and honey in the fall (positive slopes). However, the size of this benefit depended on the start date, visible in the different slopes of the blue and orange lines (Figure 4). At the earlier start date (blue lines), each additional kg of honey equivalent/supplemental feeding produced 1061 more adult bees and 4.4 more kg honey on average. At the later date (orange lines), these values decreased to 306 more adult bees and 2.5 more kg honey on average. If bees were not fed at all prior to 21 October, colony conditions were below all those graphed in the present study (Supplemental Data, Figure S16).

The start date of supplemental feeding also impacted fall colony conditions. At the middle supplemental sugar amount of 4.7 kg honey equivalent/feeding, each additional day that the supplemental feeding was delayed meant 105 fewer adult bees and 0.3 less kg honey on average in the fall (difference between blue and orange lines). Start delays made a bigger difference when more sugar was provided. Among these combinations, the largest adult bee population was achieved by beginning supplemental feeding in August (topmost point in upper plot), whereas the largest honey store occurred by beginning in September (topmost point in lower plot). Timing mattered even when the same total amount of sugar was fed over the full supplemental feeding period (visualized by total in Supplemental Data, Figure S9).

More sugar supplied during treatment feedings led to more adult bees and honey stores in the fall. At these graphed median initial conditions, the greater treatment feeding level produced 2950 more adult bees and 4.1 kg more honey on average in the fall (difference between solid and dotted lines in Figure 4). This was true regardless of supplemental feeding details, but was impacted by initial honey amount.

**Figure 5:**
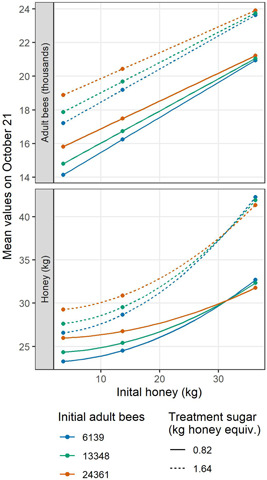
Mean effects of initial condition aspects on fall colony metrics from the linear mixed‐effects models, based on the targeted set of BEEHAVE model simulations. These values are set at the first supplemental feeding start date and 4.7 kg of honey equivalent/supplemental feeding, as an example.

The mean effects of initial honey stores and initial numbers of adult bees are the focus in Figure 5, in which supplemental feeding aspects were kept constant (23 August start date and 4.7 kg honey equivalent/feeding as an example). The slopes of all lines are positive, indicating that simulated colonies beginning with larger honey stores produced more adult bees and honey in the fall. However, the slopes vary both by the number of initial adults, and by the amount of sugar provided during treatment feeding. The number of initial adult bees was less important when the colony had more initial honey: at the 5th percentile of initial honey, there were 1674 more fall adults and 2.7 kg more fall honey between the lowest and highest shown initial adult numbers (compare among colors); however, at the 95th percentile of initial honey, there were only 274 more adults and 0.9 kg less fall honey. This reversal in relative direction among initial fall bee numbers at high inital honey levels might be reflective of the precision of the modeling, and negligibly different in more realistic settings.

The size of the effect of more initial honey on resulting fall honey depended on the amount of sugar in treatment feedings (Figure 5). Although all colonies benefitted from additional treatment‐period sugar, those beginning with more honey subsequently had considerably larger fall honey stores (difference between solid and dotted lines). For example, at the 5th percentile of initial honey, increasing the treatment sugar level grew fall honey stores by 3.3 kg and 3068 fall adults on average. However, at the 95th percentile of initial honey, this growth jumped to 9.6 kg fall honey and decreased somewhat to 2684 more adults. The change in shape from linear to quadratic was necessary to capture the considerable jump between treatment feeding levels at the 95th percentile of initial honey, further examined in the *Discussion* section.

## DISCUSSION

Because high losses of control colonies have occurred in some honey bee LSCFSs, the goal of the present study was to use data from 7 LSCFSs to identify factors that would impact control colonies' overwintering outcomes. We did so by combining and analyzing all studies' data, as well as by using honey bee colony modeling to parse out causes and effects. The results point to the importance of colony conditions at study initiation and sugar feeding amounts and timing for colony fall conditions and subsequent overwintering success. The results could be used to inform study design recommendations to decrease overwintering losses in the future.

### Analysis of data from LSCFSs

Analysis of LSCFS data showed that colonies with <4000 adult bees and very small honey stores (<2 kg) in October were the most likely to die over the winter. Colonies that primarily had larger honey stores, but also more honey/adult bee, more adult bees, and more pupae in the fall mostly survived the winter. These combined characteristics provided predictions for more than half of the colonies' overwintering survivals to >90% accuracy.

Beekeeping guidance and scientific articles report that inadequate food sources are a key cause of winter colony losses (Genersch et al. 2010; Döke et al. 2015; Tew 2015; Honey Bee Health Coalition 2019). The Honey Bee Health Coalition (2019) recommends that beekeepers leave approximately 60 pounds (27 kg) of honey in the hive for the winter, varying by latitude; Cramp (2017) recommends 15 to 30 kg for temperate climates and 40 kg for cold climates, and a beekeeper in North Carolina suggested 40 to 60 lbs (18–27 kg) of honey (Austin 2014). These values closely compare with the honey survival threshold of 30 kg for the North Carolina colonies determined in the present study. Beekeepers are also advised to only overwinter colonies that are large enough, because small fall colonies are much less likely to survive (Crowder and Harrell 2012; Döke et al. 2015; Tew 2015; Honey Bee Health Coalition 2019). More specifically, a colony needs sufficient numbers of winter bees, which later build the hive's population in the spring (Döke et al. 2015; Sanford and Bonney 2018; Honey Bee Health Coalition 2019). However, beekeepers have observed that too many bees can deplete food stores and die over the winter (Sanford and Bonney 2018), which supports our finding of a low honey‐to‐bee ratio being riskier for colony survival. Although sources also report that a colony's size of pollen reserves impact overwintering success (Döke et al. 2015; Honey Bee Health Coalition 2019), the present study did not find this association.

Factors other than honey stores and colony size are likely to influence overwintering survival, such as mites or disease (Genersch et al. 2010; Döke et al. 2015; Tew 2015; Honey Bee Health Coalition 2019), age of the queen (Genersch et al. 2010; Döke et al. 2015), characteristics of winter bees (Mattila et al. 2001; Döke et al. 2015), or water supply (Honey Bee Health Coalition 2019). During the 7 LSCFSs, the levels of *Varroa* mite and *Nosema* spores were assessed over time, and colonies were properly treated following the beekeeping recommendations. The *Varroa* and *Nosema* levels in the studies remained low, with no apparent correlation with overwintering success (e.g., the mean of all *Varroa* measurements was 1.4 mites/100 bees). Also, all queens were of comparable age in these studies. Other factors may contribute to the limitations of the identified thresholds from the LSCFSs in the present study. These thresholds do not provide predictions for 44% of colonies, which had mixed results. Some of the suggested thresholds have little margin for variability, in that relatively small shifts could reduce prediction accuracy. Also, these thresholds are specific to these LSCFSs, and are unlikely to be applicable elsewhere to the precision reported in the present study. Instead, the general trends should be used to inform study design choices.

The reasons that colonies reached the fall with stronger or weaker colony conditions could not be derived from the empirical data, because the LSCFSs varied in multiple aspects related to environmental conditions and beekeeping activities. Instead, we systematically varied these aspects using the BEEHAVE model, allowing us to isolate characteristics that would impact fall colony conditions.

### BEEHAVE simulations

Initial simulations demonstrated that ranges of weather and landscape composition in the available LSCFSs were not very influential to fall colony measures. This does not suggest that these 2 aspects of environmental conditions do not broadly have an impact on honey bee colonies, but rather that their variability was too low among these 7 studies to affect different outcomes. Between the 2, weather appears to have had a relatively greater impact on fall colony condition. Substitution of year for apiary in the LME models produced highly similar results, suggesting that weather year was approximately as explanatory as apiary. Furthermore, in targeted simulations, the only 2 years with early deaths were those with the fewest foraging hours between placement in the study apiary and the first treatment feeding. Other studies in Austria and Luxembourg reported that more honey bee colony losses were associated with warmer winters and cooler Julys (Switanek et al. 2017; Beyer et al. 2018). Associations with precipitation were more mixed: more precipitation meant fewer losses in most months in Austria (Switanek et al. 2017), but the directionality of effect varied by month in Luxembourg (Beyer et al. 2018).

Although landscape might be the least explanatory factor for fall colony condition in our BEEHAVE simulations, there are considerable limitations to the applicability of this finding. Importantly, the variability in landscapes among these 7 LSCFSs was relatively small by design. All studies were conducted in the same region of North Carolina, and apiary locations were chosen to minimize mass‐flowering crops in the surrounding landscape. Land covers were dominated by deciduous forest and had relatively little agriculture within 1.5 km (mean 6%, maximum 20%). In addition, the sugar feedings partially masked the importance of bee resource availability in the landscape. In other studies, land covers such as uncultivated land and pastures have been shown to be important to honey bee colony survival when one is comparing more widely differing landscapes (Smart et al. 2016; Kuchling et al. 2018). Floral resources are the preferred sources of nectar for bees to garner sufficient honey stores for overwintering, with supplemental feeding recommended as a substitute when needed (Döke et al. 2015; Honey Bee Health Coalition 2019).

In contrast to weather and landscape composition, factors that can be controlled as part of the study design were highly influential on fall colony condition in our simulations. These factors included colony feeding patterns and the initial setup of the colonies. Providing more sugar during treatment feeding (beginning within 1 wk of arrival at the study apiaries) and supplemental feeding (later in the summer/fall) consistently led to stronger fall colonies. During the supplemental period, the benefit was even greater when feedings began earlier. When the same total amount of sugar was provided to bees starting earlier (more feedings starting in August with less sugar each), simulated colonies had more adult bees, but comparatively less fall honey; starting later (fewer feedings starting in September with more sugar each) in contrast produced comparatively fewer adult bees, but more fall honey. This suggests that feeding while the colony is still raising brood produces more adult bees in the fall, coupled with more honey consumption. Beginning feeding later avoids some population increase, but increases honey stores for the winter. It may be preferable to increase October honey via September supplemental feeding, provided that adult bee numbers are not too few, because it was the high honey threshold derived from LSCFS data that explained the most surviving colonies regardless of the number of adult bees. Although in some studies, feedings began in late October, this late start might not be effective in helping colonies overwinter. Also, although the quantity of supplemental sugar actually consumed/colony was unavailable in most studies, having these quantities in future LSCFSs would facilitate our understanding the role of supplemental feeding in overwintering survival.

Although more treatment‐period sugar always resulted in larger simulated fall honey stores, the size of this benefit depended on the initial honey amount. Colonies that began with large honey stores experienced a much larger boost from more treatment‐period sugar than did colonies with smaller initial honey stores. Based on examination of time lines in BEEHAVE, this occurred because of colony dynamics in July and August. Colonies with high initial honey already had many adult bees by mid‐July. However, when these colonies were provided less treatment sugar, their honey stores notably dropped in August. The difference between the 2 treatment‐feeding amounts therefore occurred because the high‐initial‐honey colonies grew quickly, and had greater consumption needs earlier in the season that were not met with the lesser treatment amount. Only without sufficient sugar, these large colonies' honey stores decreased in August, followed by a population decrease in September. In all cases, however, more treatment sugar strengthened fall colony measures.

These results from BEEHAVE simulations were compared with the original LSCFS data to corroborate observed trends (see the Supplemental Data, Section 6.0). The most clearly visible trend was that studies with early and higher supplemental feeding amounts had the most fall honey. Also, the results of the initial simulation set provided some comparative insight. The 2 feeding schedules that produced the largest simulated honey stores were those with the earliest start dates and the most sugar in supplemental feedings. In contrast, the 2 feeding schedules that produced the least fall honey and adult bees began supplemental feeding after 21 October. For beekeepers, timing of supplemental feeding is recommended to begin during summer or fall, or before temperatures decrease below 50 °F (10 °C; Tew 2015; Honey Bee Health Coalition 2019), supporting our findings that feeding is beneficial if it occurs in August and September rather than later. Recommendations for the amount of sugar to provide colonies depend on the size of their honey stores (Döke et al. 2015; Cramp 2017). Whether this honey‐dependent feeding recommendation from the literature should be implemented in LSCFS designs should be carefully considered; consistency is important, and regulatory authorities may raise concerns that supplemental feedings can potentially reduce exposure to stored residues from treatment feedings and mask reduced foraging effects. We believe these concerns can be addressed by an earlier exposure period (i.e., spacing the period between treatment and supplemental feedings prior to winter).

Colonies in the LSCFSs are assembled from bee packages several weeks prior to study initiation, allowing for control of the starting conditions of study colonies. In the present analysis, the conditions of a colony at the time of study initiation were indicative of its condition in the fall. Initial honey was clearly the dominant initial condition, making the most difference to both fall honey stores and fall adult bees. Colonies that began with more honey fared best. To a lesser extent, having more initial adults also helped, especially for colonies with less initial honey. Colonies assigned the lowest initial honey levels in BEEHAVE were the only group with a chance of dying in the summer, especially when combined with more adult bees (too many mouths to feed). The initial number of pupae somewhat improved fall colony conditions as well, although by a smaller margin on average. Initial amounts of pollen and brood had comparatively negligible effects.

As part of the design of each LSCFS, colonies were placed into 12 different apiaries. Within each apiary were 2 control colonies, paired based on similar initial conditions to ensure comparability. The choice of these initial conditions varied by study, that is, some studies paired colonies by numbers of adult bees, whereas others paired colonies by numbers of brood. The results of this BEEHAVE simulation analysis suggest that the basis of these colony pairings, as well as the choice of colonies to include in LSCFSs, should instead be based mainly on initial honey amounts. Colonies beginning with very little honey (e.g., ≤3.6 kg) potentially should be excluded. Among the remaining colonies with median or low (but >3.6 kg) initial honey levels, those with more initial adults, followed by more initial pupae, should be favored to enable growth of more successful colonies over the season.

In a comparison of these results with the original LSCFS data (see Supplemental Data, Section 6.0), apiaries with colonies that began with larger honey stores generally had larger fall colonies and fall honey stores. Initial adult bee numbers were not as consistently related to fall colony metrics; this is expected based on the smaller effect sizes determined from the simulation results. Guidance for beekeepers recommends that early in the warm season, small colonies should either be fed to grow their populations or combined with other colonies to increase their likelihood of survival (Crowder and Harrell 2012; Döke et al. 2015; Honey Bee Health Coalition 2019). This supports the present finding that initially small colonies can grow over the season if they have enough honey.

The BEEHAVE simulations provide a systematic and controlled method to test the impact that changes may have on honey bee colonies. However, the model is a simplification of the real system: not all possible factors and variables influencing colonies are explicitly represented. Accordingly, the simulation results presented in the present study should not be used to make precise predictions of conditions in individual colonies. Instead, they can elucidate the relative importance of different factors and their interactions. Furthermore, the derived qualitative and categorical recommendations to improve the study design are based on data from 7 LSCFSs in North Carolina in recent years. Extrapolation to other conditions, especially other climatic regions, would require corresponding data from different studies. Specific study timing and related recommendations may be particularly dependent on climate or region.

## CONCLUSIONS

Despite their high cost and continued use in regulatory risk assessments, no formal regulatory protocol exists for conducting LSCFSs. Therefore, LSCFSs currently are not fully standardized in all aspects and can suffer from high winter losses in untreated controls in some cases. With systematic simulations, we derived recommendations for study design elements that could lead to higher likelihood of overwinter survival of control colonies. These recommendations should be considered alongside local best beekeeping practices (e.g., following recommendations of the local apiary inspectors). The following recommendations could be used to inform the standardization of LSCFS protocol. 1) Supplemental feedings are likely the most important factor for fall colony condition, among the factors evaluated: provide more supplemental sugar to increase honey store and colony size before the winter. 2) Begin supplemental feeding no later than August or September; beginning later (September) can increase fall honey stores more; beginning earlier (August) can increase fall colony sizes more. 3) Provide more treatment sugar to increase fall colony size and honey stores; beginning treatment feeding shortly after placement in apiaries can help prevent early colony losses for those starting with smaller honey reserves. 4) Choice of colonies to include in LSCFSs, as well as their organization within apiaries, should be mainly based on size of initial honey stores; colonies with large honey stores are likely the strongest, regardless of number of bees; colonies with small initial honey stores (e.g., approximately ≤4 kg) are at risk of early death during the summer, particularly if they have comparatively more adult bees (more mouths to feed), and should be excluded from studies. 5) As a secondary criterion at the initial placement stage, avoid colonies that have a combination of medium‐to‐low honey and low adults. In contrast, colonies with honey stores approximately ≤14 kg (but >4 kg) can grow prior to the winter if they have more initial adults.

The application of BEEHAVE to inform study design of field studies such as LSCFSs presents a valuable contribution to honey bee risk assessments. Because the studies are resource and time intensive, systematic field testing of design options is not feasible. At the same time, the success of the studies should be ensured and is strongly dependent on the health and overwintering survival of the untreated control colonies. Beyond the recommendations for the study design of LSCFSs, our findings demonstrate how ecological models could be applied more generally to assess the importance of multiple factors in complex studies and inform designs of extensive studies for a specific purpose.

## Supplemental Data

The Supplemental Data are available on the Wiley Online Library at https://doi.org/10.1002/etc.4844.

## Supporting information

This article includes online‐only Supplemental Data.

Supporting information.

## Data Availability

Data, associated metadata, and calculation tools are available from the corresponding author (abi-akarf@waterborne-env.com).
