# Supplementary material for: Simulating Honey Bee Large‐Scale Colony Feeding Studies Using the BEEHAVE Model—Part II: Analysis of Overwintering Outcomes
Source: Environ Toxicol Chem. 2020 Sep 22;39(11):2286–97. doi: 10.1002/etc.4844 (PMC7702061; doi:10.1002/etc.4844)

SUPPLEMENTAL DATA

TITLE: Analysis of overwintering outcomes in honey bee large-scale colony feeding studies using the BEEHAVE model

**Table of Contents of Supplemental Data**

[1.0 Supplemental figures and tables 2](#_Toc45872153)

[2.0 Methodology details of the initial simulations identifying factors impacting fall colony conditions 11](#_Toc45872154)

[3.0 Simulations of feeding schedules 12](#_Toc45872155)

[3.1 Methods: Simulations of feeding schedules 12](#_Toc45872156)

[3.1.1 Setup of feeding simulations 12](#_Toc45872157)

[3.1.2 Data analysis of feeding simulations 14](#_Toc45872158)

[3.2 Results: Simulations of feeding schedules 14](#_Toc45872159)

[3.2.1 Graphical comparison 14](#_Toc45872160)

[3.2.2 Statistical analysis 16](#_Toc45872161)

[4.0 Simulations of initial conditions 22](#_Toc45872162)

[4.1 Methods: Simulations of initial conditions 22](#_Toc45872163)

[4.1.1 Setup of initial condition simulations 22](#_Toc45872164)

[4.1.2 Data analysis of initial condition simulations 24](#_Toc45872165)

[4.2 Results: Simulations of initial conditions 24](#_Toc45872166)

[4.2.1 Graphical comparison 24](#_Toc45872167)

[4.2.2 Statistical analysis 25](#_Toc45872168)

[4.2.3 Binary analysis of fall losses from initial conditions 31](#_Toc45872169)

[5.0 Simulations targeting study design characteristics: Graphical comparison of results 32](#_Toc45872170)

[6.0 Comparison to relationships in LSCFS data 34](#_Toc45872171)

[6.1 Methods: Comparison to relationships in LSCFS data 34](#_Toc45872172)

[6.2 Results: Comparison to relationships in LSCFS data 35](#_Toc45872173)

# Supplemental figures and tables

Figure S1. Dates of colony condition assessments (CCA) by study. Numbers mark the CCA on the date they occurred. Some studies and CCAs spanned over multiple days across apiaries. CCAs did not occur over the winter, and the dotted line marks the start of the new year. The final fall CCA of each study, in green, was used in the more detailed fall colony condition threshold analysis. LSCFSs are labeled as “CFS_[year]_[study number]”.


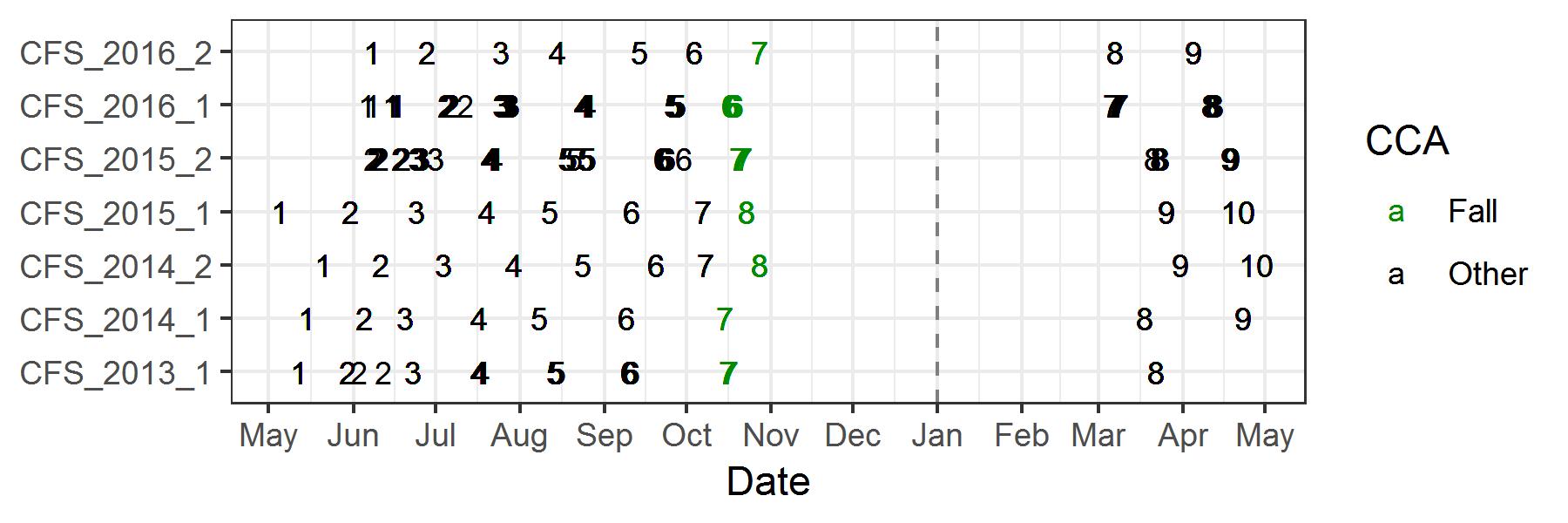


Figure S2. Graphs summarizing different aspects varying among analyzed LSCFSs. A) number of colonies that survived or were lost over the winter; B) mean land cover percentages across all apiaries; C) sum of foraging hours during June-October of each study’s first year, further explained in Schmolke et al., subm.; D) total sugar fed in the first study year, colored by feeding period; E) box plots of adult bees and honey stores when colonies were first measured in study apiaries. Colonies that died before the fall are not included.


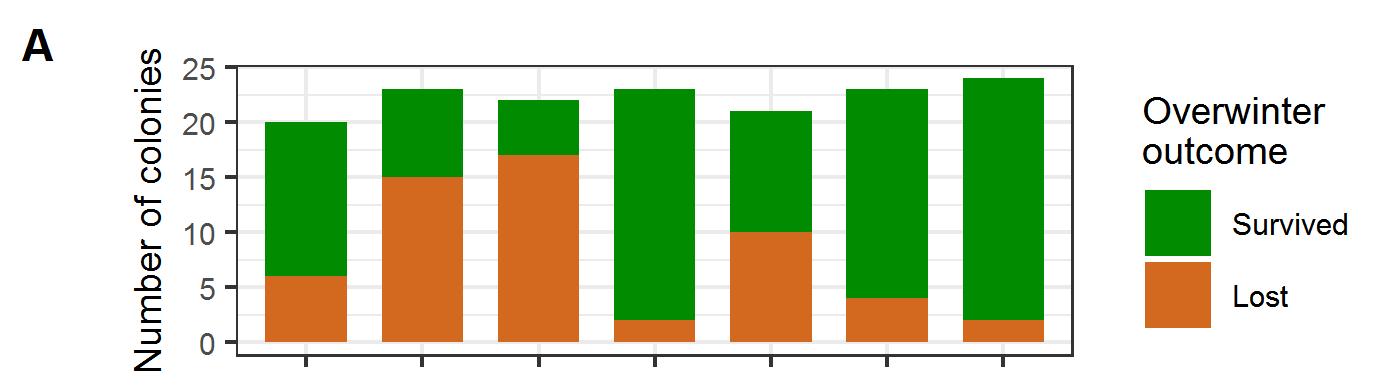

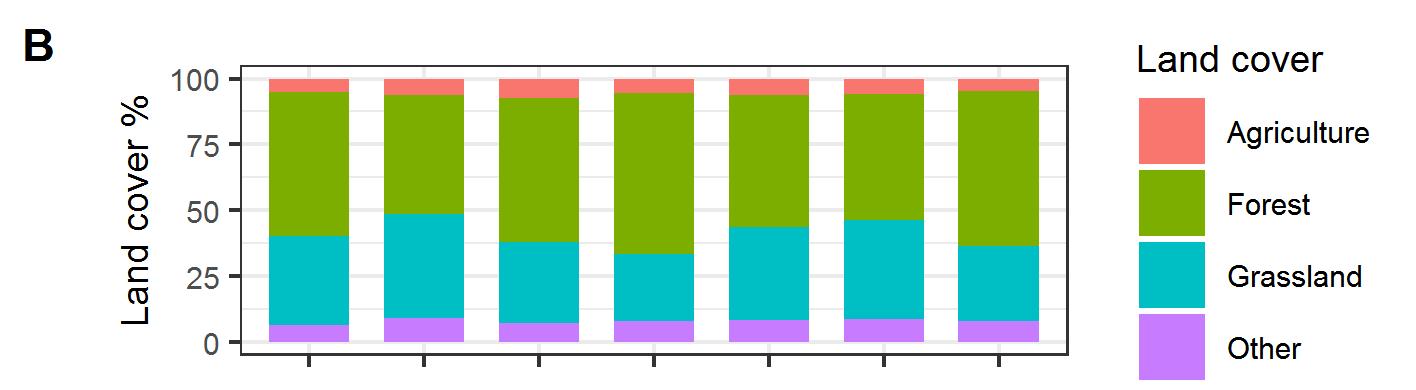

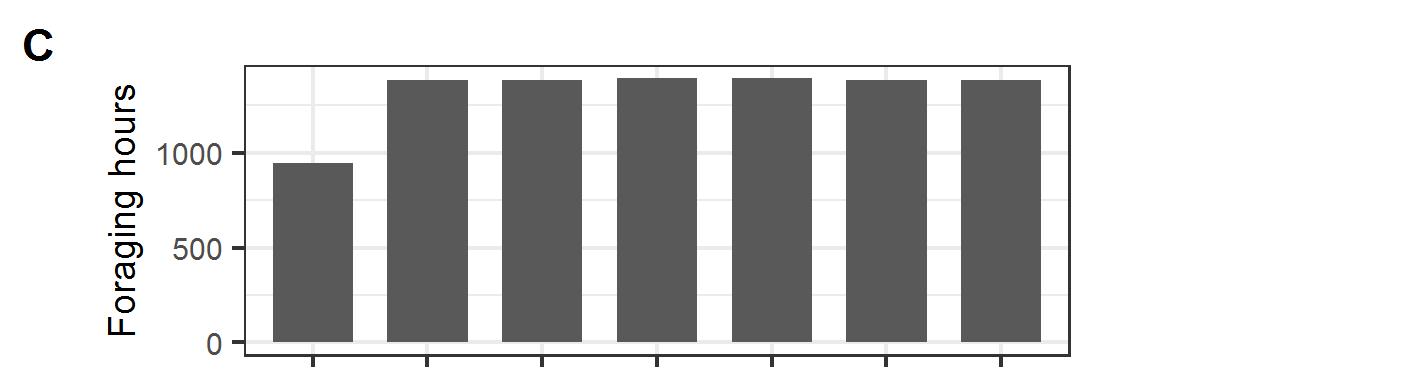

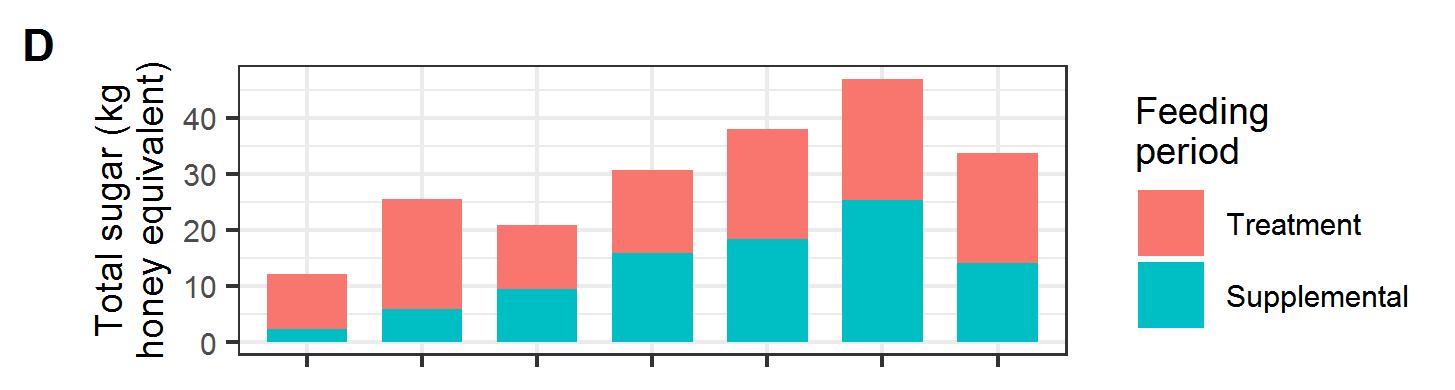

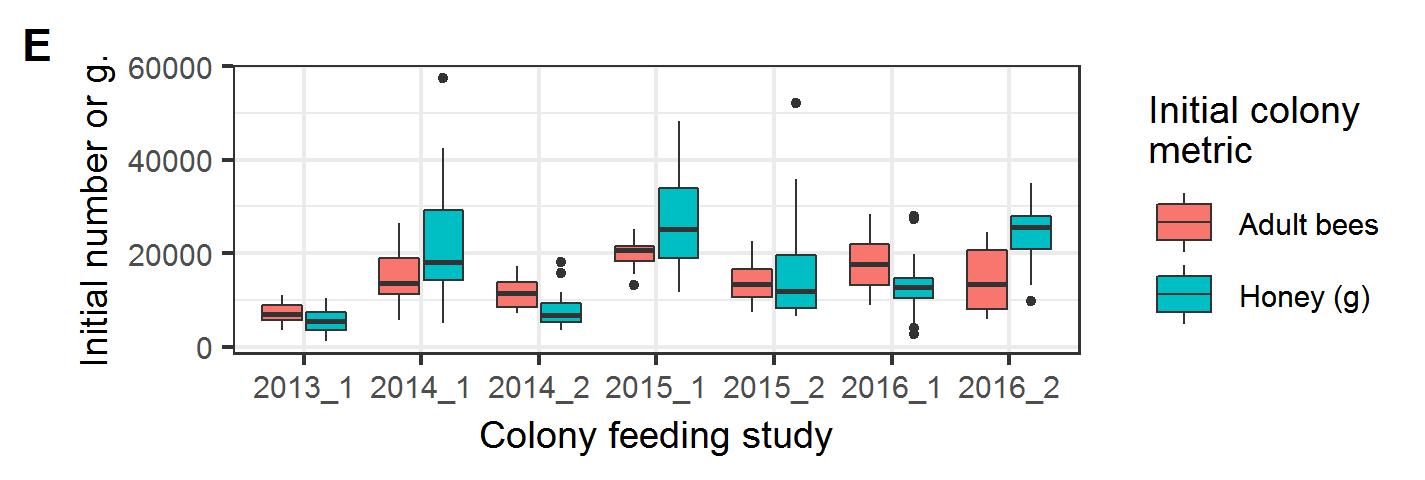


Figure S3. Actual dates and amounts of sugar fed (in kg honey equivalent) over the first year of each study, colored by feeding type. The dotted line marks October 21, the fall assessment date used in this analysis. Feeding volumes and sugar concentrations also varied, but differences were standardized by representing the sugar amount only. LSCFSs are labeled as “CFS_[year]_[study number]”.


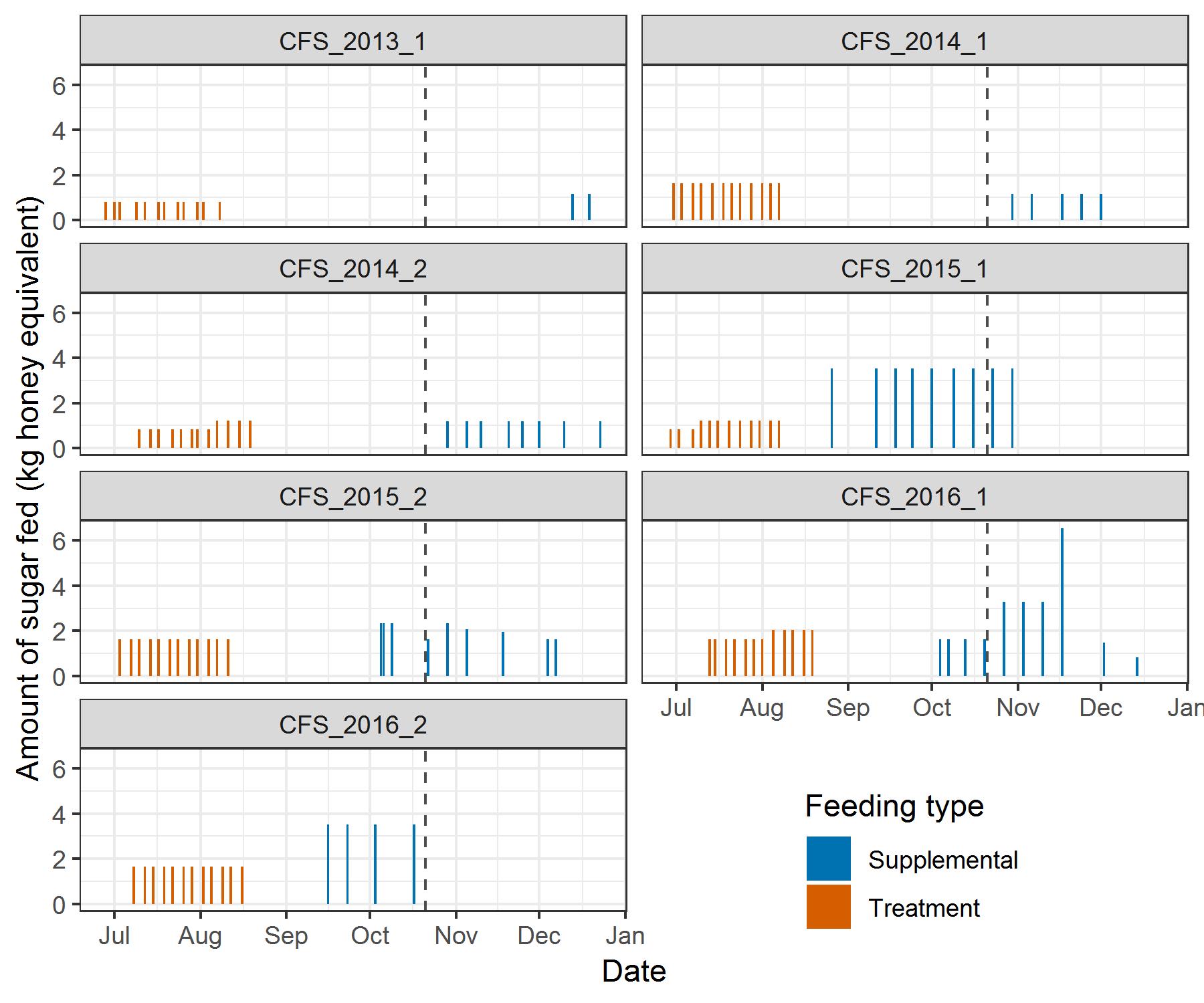


Table S1. List of the eight apiaries used in BEEHAVE simulations, spanning all studies and including a mixture of fall conditions. Overwintering results are also included for reference.

| **LSCFS** | **Apiary** | **Relative fall condition** | **Overwintering results (2 colonies per apiary)** |
| --- | --- | --- | --- |
| 2013_1 | G | Medium | Lost, surviving |
| 2013_1 | K | Lost | Lost, lost |
| 2014_1 | J | Medium | Lost, surviving |
| 2014_2 | D | Lost | Lost, lost |
| 2015_1 | L | Surviving | Surviving, surviving |
| 2015_2 | F | Medium | Lost, surviving |
| 2016_1 | K | Medium | Lost, surviving |
| 2016_2 | F | Surviving | Surviving, surviving |

Figure S4. Time line of all colonies’ adult bees (top), ratio of honey/adults on a log scale (middle), and pupae (bottom), as measured during all condition assessments in the first year of each study. Points and lines are colored by overwintering outcome. The lines are LOESS curves, with 95% confidence intervals as gray shaded areas.


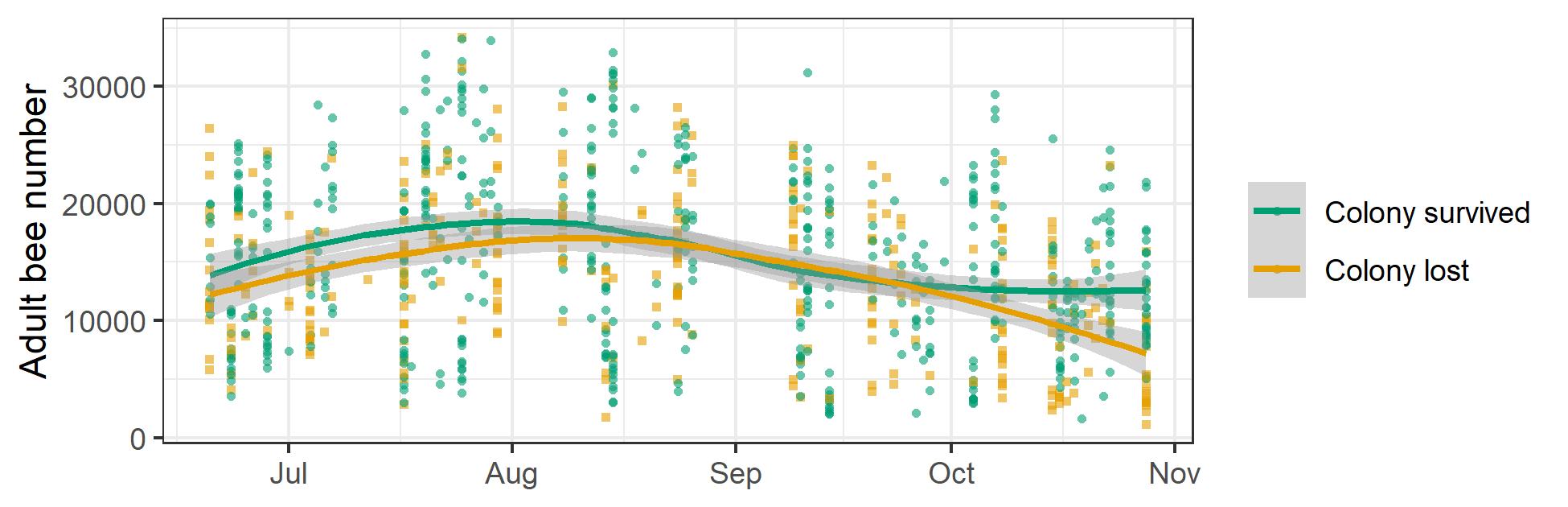


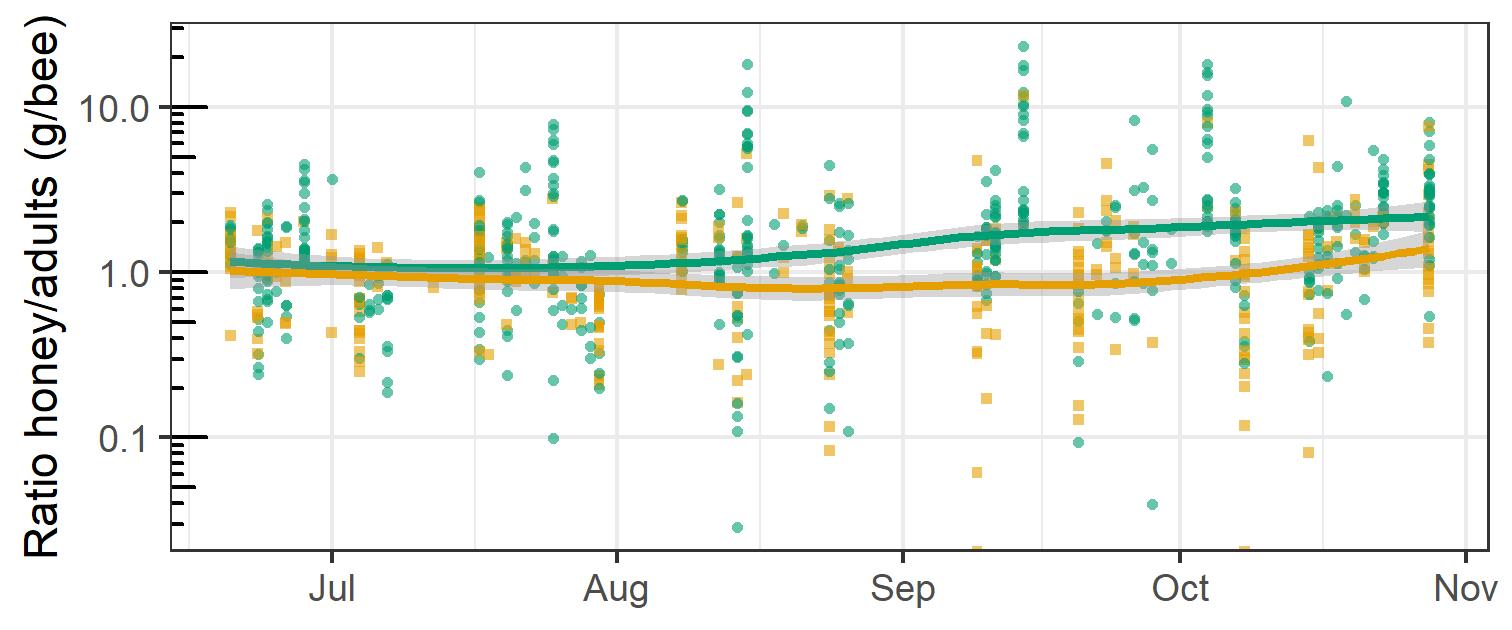


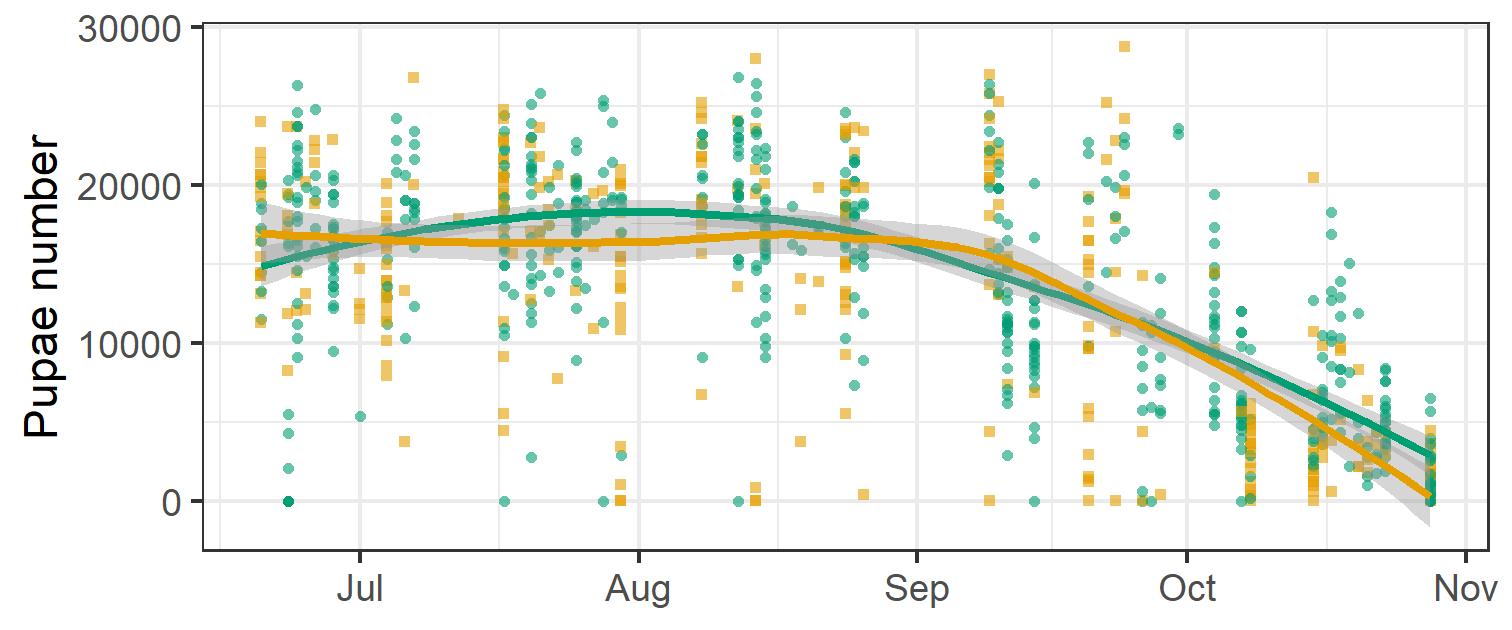


Figure S5. Box plots of all LSCFS’ colonies fall metrics classified by overwintering status and grouped by metric, as labeled. Orange dotted lines mark loss thresholds, and green dotted lines mark survival thresholds. Gray points show individual colonies’ values. All colonies are included in the top two graphs; the ratio graph excludes colonies already explained by the honey and adult bee thresholds; and the pupae graph excludes colonies explained by any of the other three. Boxes extend from the 25th to 75th percentile (interquartile range, IQR), with the central line at the median and the diamond at the mean. Whiskers extend up to 1.5x the IQR.


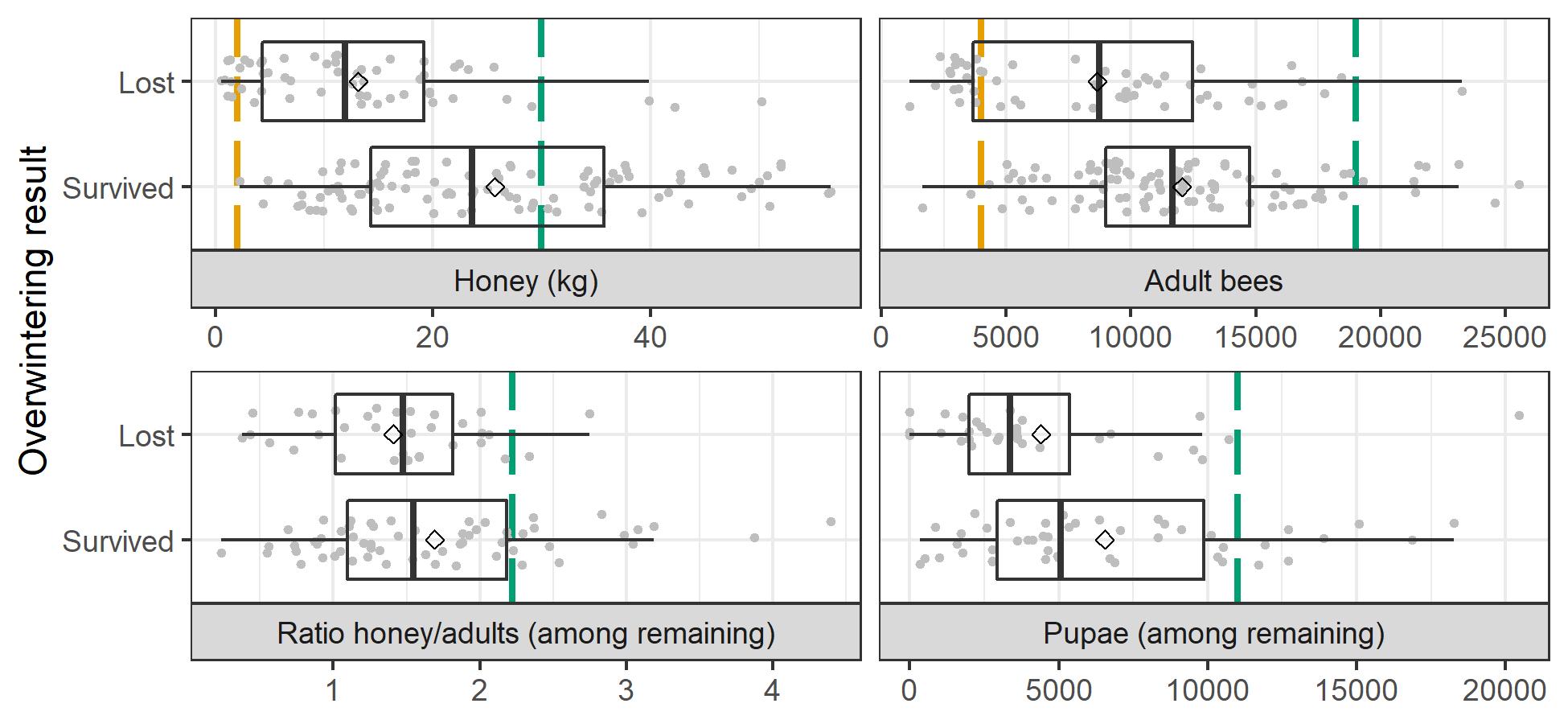


Table S2. Counts of colonies classified by actual overwintering survival/loss versus predictions from four different metrics’ thresholds. Honey and adults (top two tables) each have a survival and loss threshold, and each table includes all colonies beyond thresholds. Ratio of honey per adult bee and pupae metrics only have survival thresholds. The ratio table excludes colonies already explained by the honey and adult bee thresholds; the pupae table excludes colonies already explained by all three previous thresholds.

| **Honey** |  | Predicted | |  | **Adult bees** | | Predicted | |
| --- | --- | --- | --- | --- | --- | --- | --- | --- |
|  |  | Survival >30 kg | Loss <2 kg |  |  |  | Survival >19,000 | Loss <4,000 |
| Actual | Survived | 36 | 0 |  | Actual | Survived | 8 | 2 |
|  | Lost | 3 | 7 |  |  | Lost | 1 | 18 |
|  | Accuracy | 92% | 100% |  |  | Accuracy | 89% | 90% |
|  |  |  |  |  |  |  |  |  |
| **Ratio** |  | Predicted |  |  | **Pupae** |  | Predicted |  |
| (among remaining) | | Survival >2.22 g/bee |  |  | (among remaining) | | Survival >11,000 |  |
| Actual | Survived | 14 |  |  | Actual | Survived | 8 |  |
|  | Lost | 2 |  |  |  | Lost | 1 |  |
|  | Accuracy | 88% |  |  |  | Accuracy | 89% |  |

Table S3. Number of hypothetical scenarios that resulted in some or all replicate colony deaths before October 21, from the targeted set of scenarios. Only those with 5^th^ percentile initial honey (3.6 kg) are included, since no losses occurred at the higher amounts. The median level of initial adults produced more losses, suggesting that more bees potentially strain limited resources. The lower treatment sugar amount also produced more of these losses (not shown). The only apiaries with affected colonies were from years 2013 and 2016, which had the fewest foraging hours between placement in the study apiary and the first treatment feeding (15 hours each, compared to 59 hours in 2014 and 44 hours in 2015).

|  | 0.82 kg honey equivalent per treatment feeding | | 1.64 kg honey equivalent per treatment feeding | |
| --- | --- | --- | --- | --- |
|  | Initial adult bees | | Initial adult bees | |
| Apiary | 5^th^ percentile (6,139) | 50^th^ percentile (13,348) | 5^th^ percentile (6,139) | 50^th^ percentile (13,348) |
| CFS_2013_1-G | 8 | 8 | 0 | 8 |
| CFS_2013_1-K | 8 | 8 | 0 | 8 |
| CFS_2014_1-J | 0 | 0 | 0 | 0 |
| CFS_2014_2-D | 0 | 0 | 0 | 0 |
| CFS_2015_1-L | 0 | 0 | 0 | 0 |
| CFS_2015_2-F | 0 | 0 | 0 | 0 |
| CFS_2016_1-K | 0 | 8 | 0 | 8 |
| CFS_2016_2-F | 0 | 8 | 0 | 8 |

Figure S6. Residual plot of the LME model of fall adult bees from the targeted simulations. BEEHAVE output is on the X axis, and LME-model output on the Y axis. Points are colored by apiary, and are shown with random-effects shifts. The black diagonal line is 1:1, representing a perfect prediction. Apiaries are labeled as “CFS_[year]_[study number]-[apiary ID]”.


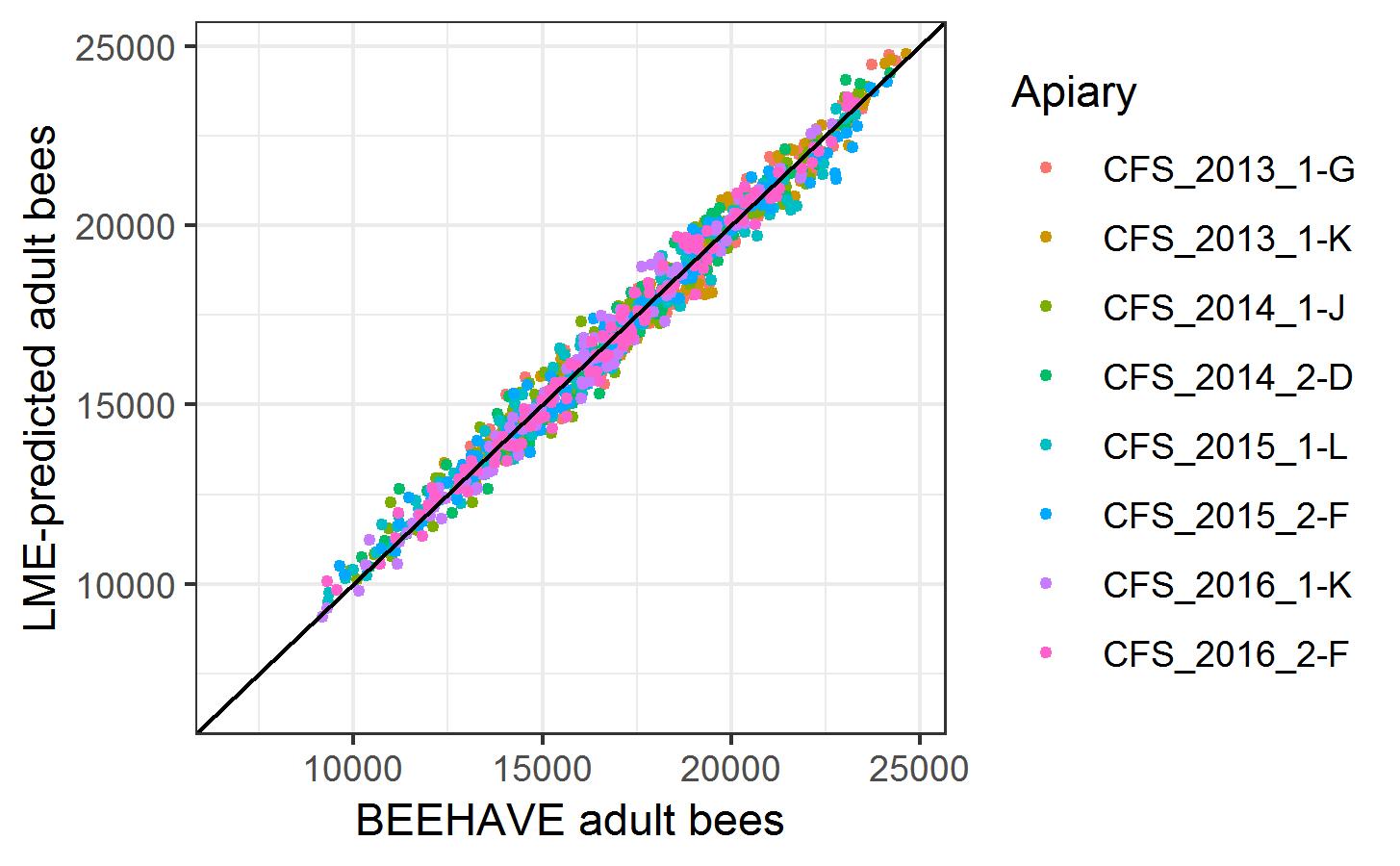


Figure S7. Residual plot of the LME model of fall honey stores from the targeted simulations. BEEHAVE output is on the X axis, and LME-model output on the Y axis. Points are colored by apiary, and are shown with random-effects shifts. The black diagonal line is 1:1, representing a perfect prediction. Apiaries are labeled as “CFS_[year]_[study number]-[apiary ID]”.


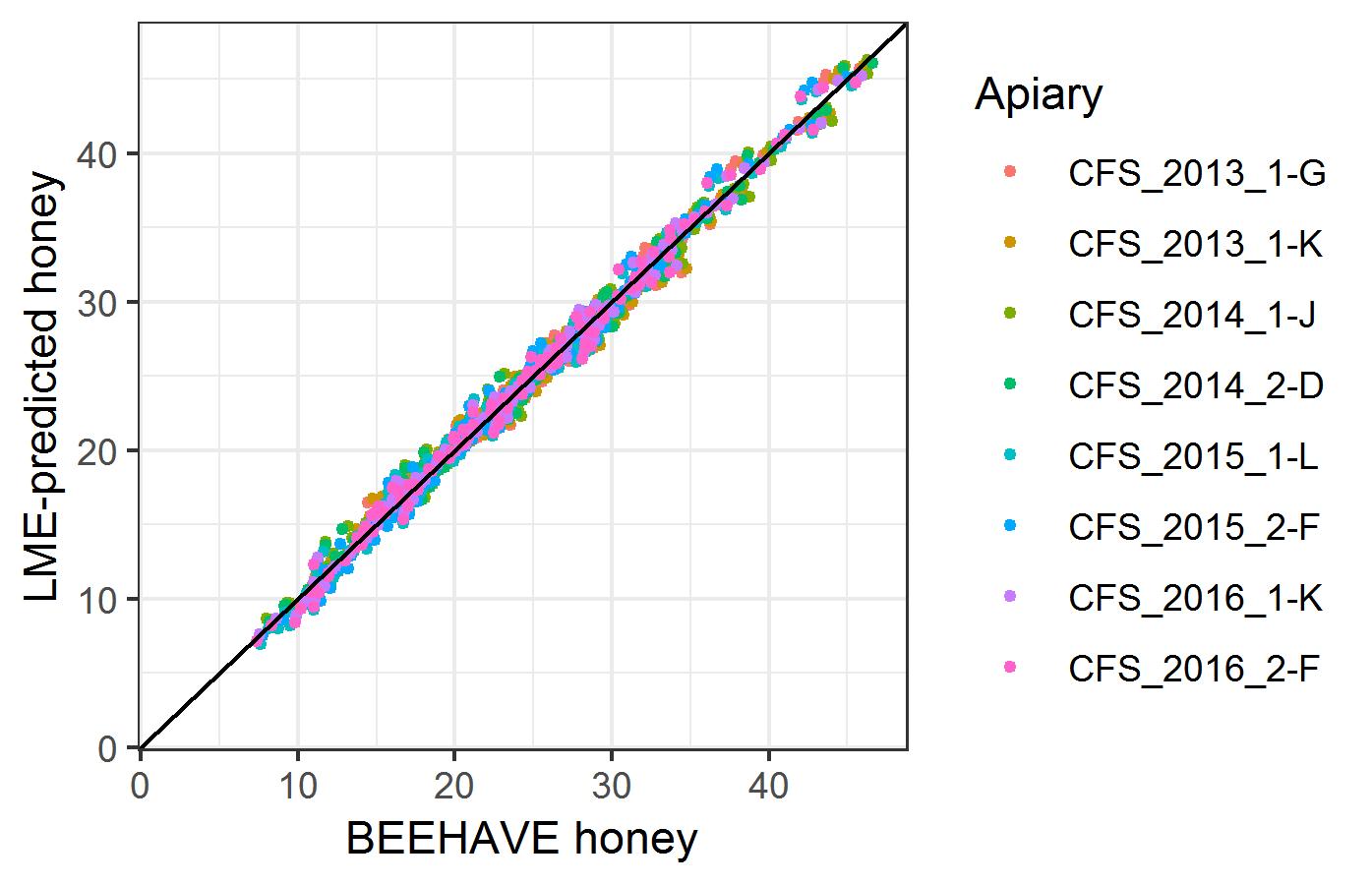


Figure S8. Histograms of residuals from the honey LME model of the targeted simulations, split by apiary and colored by amount of initial honey. The dotted line marks 0, a perfect prediction. Distributions are fairly normal and colors are fairly evenly split, due to the interaction between the treatment feeding and initial honey variables. Predictions by apiary appear balanced in over- and under-prediction, indicating that this model has captured between-apiary differences. Apiaries are labeled as “CFS_[year]_[study number]-[apiary ID]”.


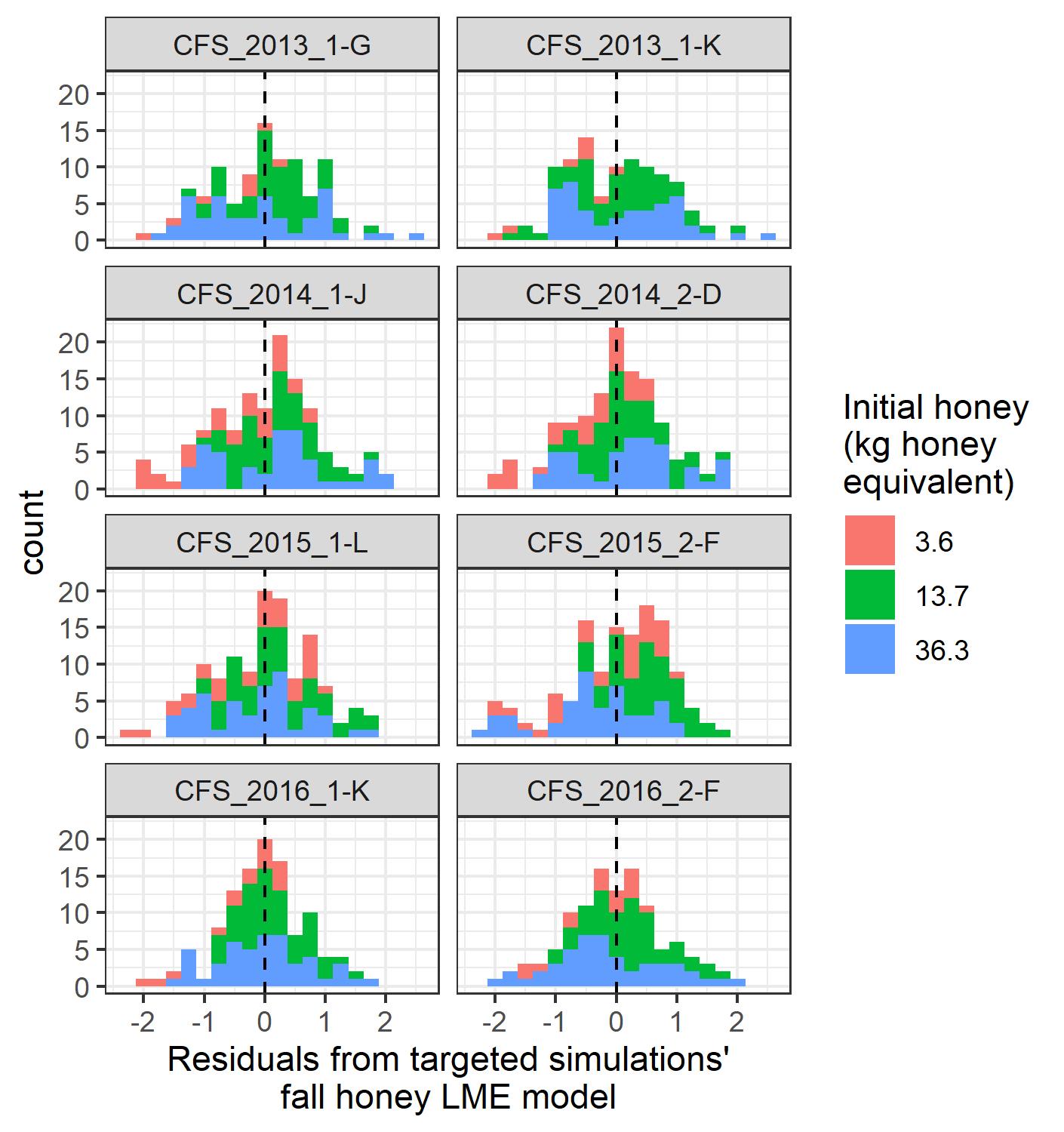


Figure S9. Average effects of feeding schedule aspects on fall colony metrics from the LME models, based on the targeted set of BEEHAVE simulations. The left graph shows adult bees on the Y axis, and the right graph shows honey stores. The total sugar provided over all supplemental feedings is on the X axis, supplemental start dates vary by color, and sugar amounts per treatment feeding vary by line type as labeled. Earlier starts to supplemental feedings resulted in more adult bees and less honey, despite the same amount of sugar provided.


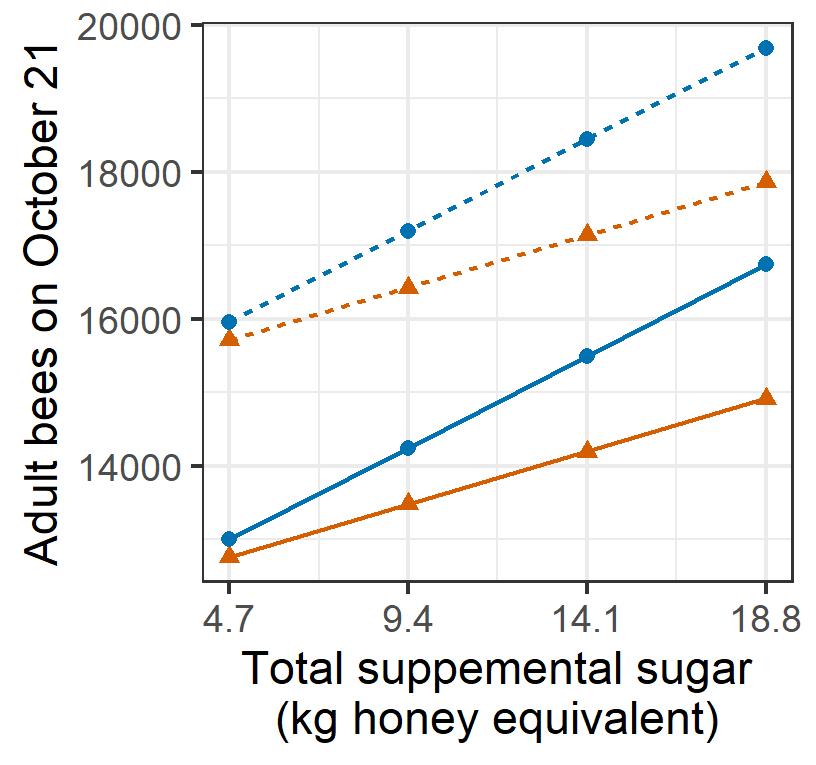

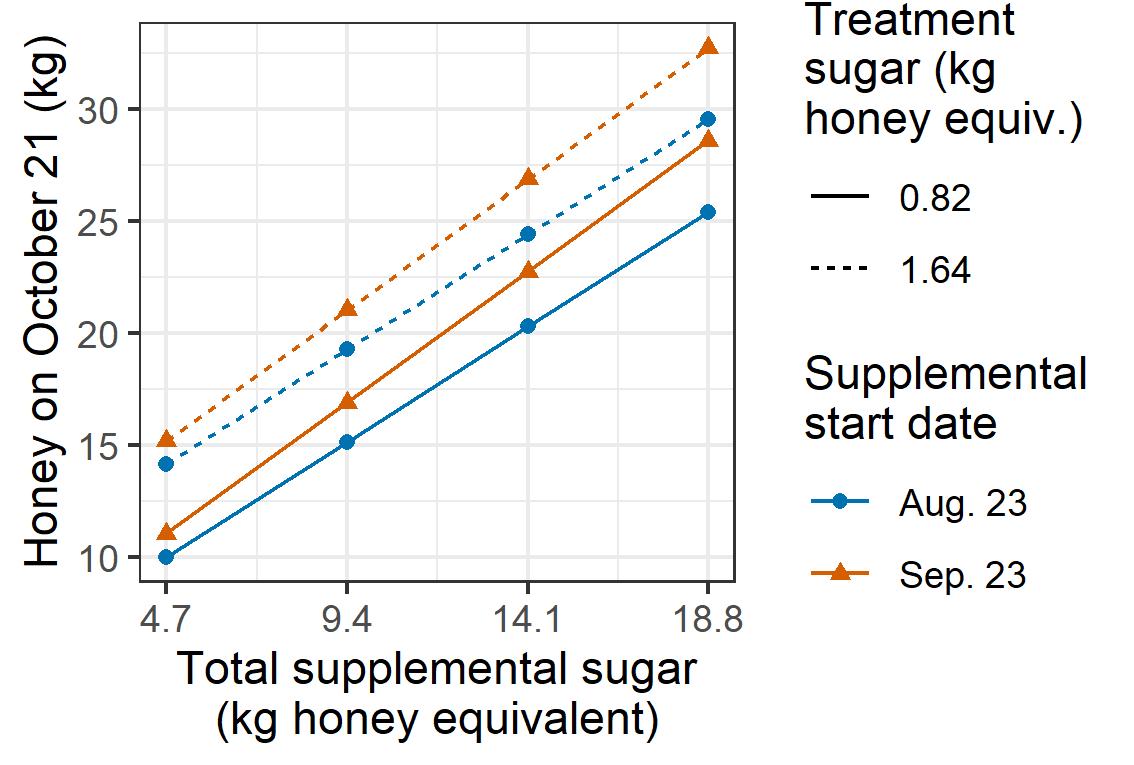


# Methodology details of the initial simulations identifying factors impacting fall colony conditions

Table S4. All factors applied in the initial BEEHAVE simulations testing variable ranges. All combinations of these factors were run.

| **BEEHAVE input type** | **Year, LSCFS, or apiary used** | **Reason** |
| --- | --- | --- |
| Weather | 2013 | All studies’ weather years applied |
|  | 2014 | All studies’ weather years applied |
|  | 2015 | All studies’ weather years applied |
|  | 2016 | All studies’ weather years applied |
| Feeding schedule | CFS_2013_1 | All studies’ feeding schedules applied |
|  | CFS_2014_1 | All studies’ feeding schedules applied |
|  | CFS_2014_2 | All studies’ feeding schedules applied |
|  | CFS_2015_1 | All studies’ feeding schedules applied |
|  | CFS_2015_2 | All studies’ feeding schedules applied |
|  | CFS_2016_1 | All studies’ feeding schedules applied |
|  | CFS_2016_2 | All studies’ feeding schedules applied |
| Initial colony conditions | CFS_2013_1-L | Fewest adult bees |
|  | CFS_2016_1-A | Most adult bees |
|  | CFS_2013_1-J | Fewest pupae |
|  | CFS_2014_1-A | Most pupae and 2^nd^-largest honey store |
|  | CFS_2016_1-F | Smallest honey store |
|  | CFS_2014_1-I | Near-average adult bees, pupae, and honey store |
| Landscape | CFS_2014_1-J | Least forest cover |
|  | CFS_2016_2-L | Most forest cover; least grass/pasture cover |
|  | CFS_2016_1-G | Least cover of agricultural fields |
|  | CFS_2015_2-C | Most cover of agricultural fields |
|  | CFS_2014_1-F | Most grassland/pasture cover |

One year-specific weather input file from a single station was used for each of the study years due to proximity of apiaries; all years were included (US NOAA 2018). Since each of the seven studies had a unique feeding schedule, all seven were applied. To represent the different initial colony conditions, the mean of initial adult bee, pupae, and honey store values from the two control colonies within each apiary were calculated. Using the mean acknowledges the inherent variability in response between similar colonies in the same location. Based on these distributions of control means across all studies, apiaries were chosen to represent the upper and lower ends of each metric, as well as an apiary with near-average values. Similarly, landscape variability was represented by compiling proportions of forest, grassland/pasture, and agricultural land covers within 1.5 km around all apiaries. Apiaries were chosen to represent the upper and lower ends of each. *Varroa* mites and *Nosema* infestations were not simulated because mites do not affect simulated colonies in BEEHAVE unless they transmit a virus, *Nosema* is not represented in BEEHAVE, and these data in the LSCFSs were infrequent.

With apiaries representing four weather years, seven feeding schedules, six initial conditions, and five landscapes, 840 combinations were produced. This systematic approach ensured that each simulation varies in only one aspect, thus isolating cause and effect. To add the element of random variability, ten repetitions of each combination were run, totaling 8,400 runs. From the output of these simulations, the mean number of adult bees and size of honey stores on October 21 among the sets of ten replicates were calculated.

*Reference:*

US National Oceanic and Atmospheric Administration. 2018. Data tools: Find a station. Accessed 2018 April 11. Available from: <https://www.ncdc.noaa.gov/cdo-web/datatools/findstation>.

# Simulations of feeding schedules

## Methods: Simulations of feeding schedules

First, the timing of feeding and amount of sugar supplied in each study were examined and compared, then were systematically varied to produce a set of hypothetical feeding schedules used as inputs to BEEHAVE. Second, the model outputs in the fall were graphed and statistically analyzed to link the effects of the different feeding choices to resulting fall colony conditions. These steps are detailed in this section.

### Setup of feeding simulations

Feeding schedule characteristics were simplified into a representative range of dates and sugar amounts, listed in Table S4. Since treatment feedings across all studies occurred within one week of hives’ placement within the study apiary, each apiary was assigned one of four start dates, one week apart (June 22-July 13), based on their study initiation date (see Schmolke et al., subm. and Figure S1).

Table S5. Feeding schedule aspects systematically varied and run through BEEHAVE. All combinations of these aspects produced 36 hypothetical feeding schedules.

| **Feeding period** | **Aspect** | **Number of levels** | **Levels applied** |
| --- | --- | --- | --- |
| Treatment | Start date | 1 | One date per apiary, based on reset date: 2014_1J assigned June 22, 2014_2D July 6, 2016_1K July 13, and all remaining apiaries June 29 (Figure S10) |
| Treatment | Amount of sugar fed | 2 | 0.82 or 1.64 kg honey equivalent of sugar per feeding |
| Supplemental | Start date | 3 | August 23, September 23, or none prior to fall analysis date |
| Supplemental | Amount of sugar fed | 6 | 1.18, 2.35, 3.53, 4.70, 5.88, or 7.05 kg honey equivalent of sugar per feeding |

Figure S10. Dates of each simulated apiary’s first CCA after placement, which was the date at which BEEHAVE was reset to measured values (points), and the first day of treatment feedings as applied in the BEEHAVE feeding simulations (vertical lines). The four applied dates were used for simplicity, then assigned to apiaries by CCA date to fall within one week. Apiary CFS_2014_1_J was assigned June 22, CFS_2014_2_D July 6, CFS_2016_1_K July 13, and all remaining apiaries June 29. Apiaries are labeled as “CFS_[year]_[study number]_[apiary ID]”.


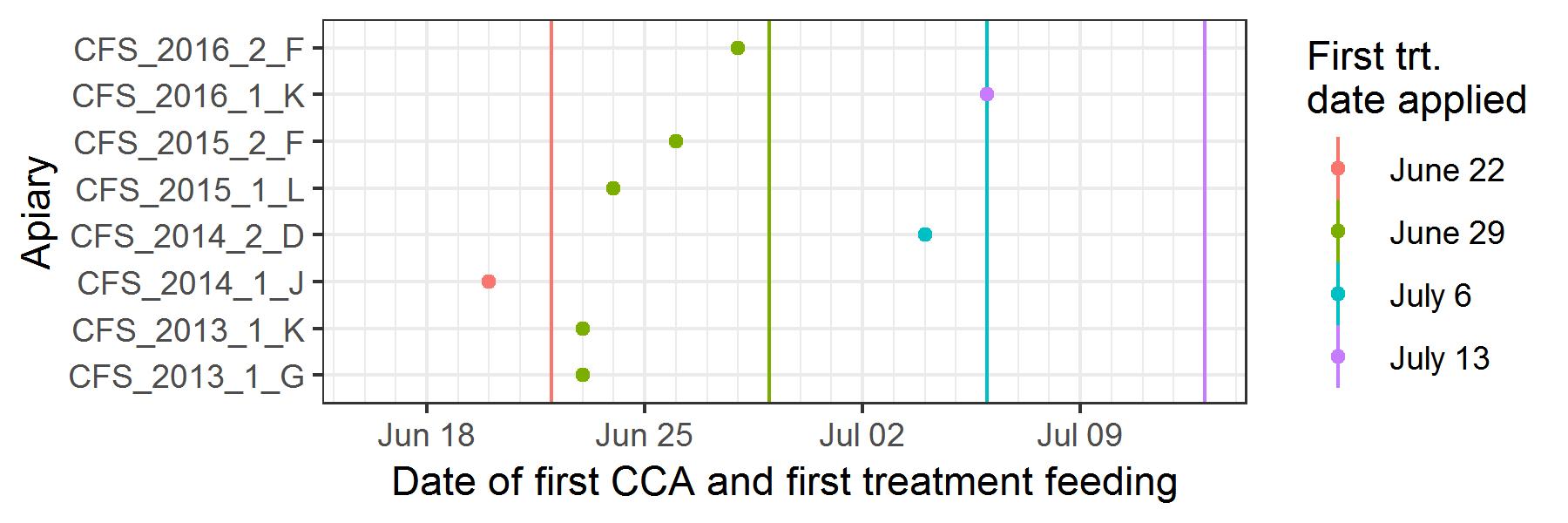


All simulations had 12 treatment feedings thereafter, 3 to 4 days apart. Each hypothetical feeding scenario was assigned either 0.82 or 1.64 kg honey equivalent (respectively corresponds to 1 or 2 liters of solution comprising one part sugar, one part water by weight) per treatment feeding. Supplemental feedings began at a wider range of dates across studies, so the three simulated starting dates were August 23, September 23, and none prior to the fall date of focus in this analysis, October 21. No supplemental feedings in the simulations reflected the start of supplemental feedings after the October 21 cut-off date, which occurred in three of the seven studies. To represent these in the data analysis, they were modeled as having occurred on October 23. All supplemental feedings were applied on the 8th and 23rd of each month but ended on October 8 regardless of the start date. As such, the hypothetical scenarios beginning August 23 were fed four times, and those beginning September 23 were fed twice in BEEHAVE. Supplemental feedings also varied considerably in the amount of sugar per feeding; six levels between 1.18 and 7.05 kg honey equivalent were used, each 1.175 kg apart.

All combinations of these feeding aspects were applied to eight different apiaries, which were chosen to span studies, and to vary by fall condition and overwintering success of the control colonies. All other unique aspects of these apiaries that varied in BEEHAVE – their weather, surrounding landscape, and initial conditions – were used as-is, such that the only modification was their assigned hypothetical feeding scenario. Applying the same set of feeding scenarios to multiple apiaries allows for evaluation of the consistency of response to feeding schedules applied in different circumstances, and consideration of remaining unexplained variability after controlling for feeding schedules.

The two treatment feeding amounts, three supplemental feeding start dates, and six supplemental feeding amounts produced 36 hypothetical feeding schedules. Each was applied to eight apiaries, producing 288 scenarios. Each scenario was repeated 20 times to incorporate stochasticity, totaling 5,760 runs. The number of adult bees and size of honey store on October 21 was pulled from each of these runs, and the means of each set of 20 replicates were calculated, representing fall colony condition. These values served as the basis of the statistical analysis.

### Data analysis of feeding simulations

The goal of this statistical analysis was to identify whether the different timing and sugar amounts of feeding schedules impacted resulting numbers of adult bees and honey stores in the fall, and if so, to quantify those impacts. In turn, these results could be used to inform colony feeding decisions to potentially reduce overwintering losses in future studies.

Output from all hypothetical feeding scenarios were graphed to inform trends prior to statistical testing (package ggplot2, Wickham et al. 2016). The thresholds for survival and loss based on adult bees and honey stores, identified from the LSCFS data analysis, were graphed alongside the BEEHAVE output for perspective. The pupae threshold was not used because BEEHAVE-output pupae in the fall is less reflective of LSCFS data, as the model was not calibrated to pupae (Schmolke et al., subm.). In addition, brood numbers including pupae are low in October because the egg production by the queen ceases prior to overwintering. Adult bee numbers and honey stores were used as most reflective measures of colony condition prior to overwintering.

The number of adult bees on October 21 simulated with BEEHAVE was the dependent, or predicted, variable in the first statistical model, and the amount of honey in colonies on October 21 was predicted in the second statistical model. Each was set up as a linear mixed-effects (LME) model, which is a combination of least-squares multiple regression and ANOVA to account for a grouping structure in the data. The eight apiaries, which each have known inherent uniqueness, were the random-effect groups statistically represented by intercept shifts. Three independent (or fixed) variables and their interactions were tested: the amount fed during each treatment feeding as kg of honey equivalent, the first day of supplemental feeding as number of days after August 23 (the first date applied), and the amount fed during each supplemental feeding as kg of honey equivalent. Analyses were performed in R software (R Core Team 2019) with package lmerTest (Kuznetsova et al., 2017). Residual plots were checked for linearity, heteroscedasticity, and normality. A measure of goodness of fit for LME models was calculated and reported (R^2^_β_, Kenward-Roger approach; Edwards et al., 2008; Jaeger, 2017).

## Results: Simulations of feeding schedules

Characteristics of the different feeding schedules across LSCFSs were identified, systematically varied, and run through BEEHAVE as hypothetical feeding schedules. Results of the effects of scenarios’ different timings and amounts of sugar fed in both treatment and supplemental feedings are examined here graphically and statistically.

### Graphical comparison

All scenarios’ results were grouped by their applied feeding characteristics in Figure S11, in which all eight apiaries are included. The Y axis of the top row of plots shows the mean number of adult bees in the fall from each of 20 replicate runs, and the Y axis of the bottom row shows the mean fall honey stores. The left plots show scenarios with the lower amount of sugar per treatment feeding, and the right plots the higher amount. The X axis of all plots is the supplemental feeding start date, and the colors represent the amount fed per supplemental feeding. The overwintering survival thresholds of adult bees and honey that were identified from the LSCFS data above are shown as dotted lines for comparison; the loss thresholds are below all output values (not applicable).

Figure S11. Box plot of fall adult bees and honey stores output from the feeding-schedule BEEHAVE simulations. Plots are split as labeled by amount of sugar per treatment feeding and resulting fall adults/honey. Along the X axis is the first date of supplemental feeding, and colors distinguish sugar amounts provided per feeding. Dotted lines mark the overwintering survival threshold.


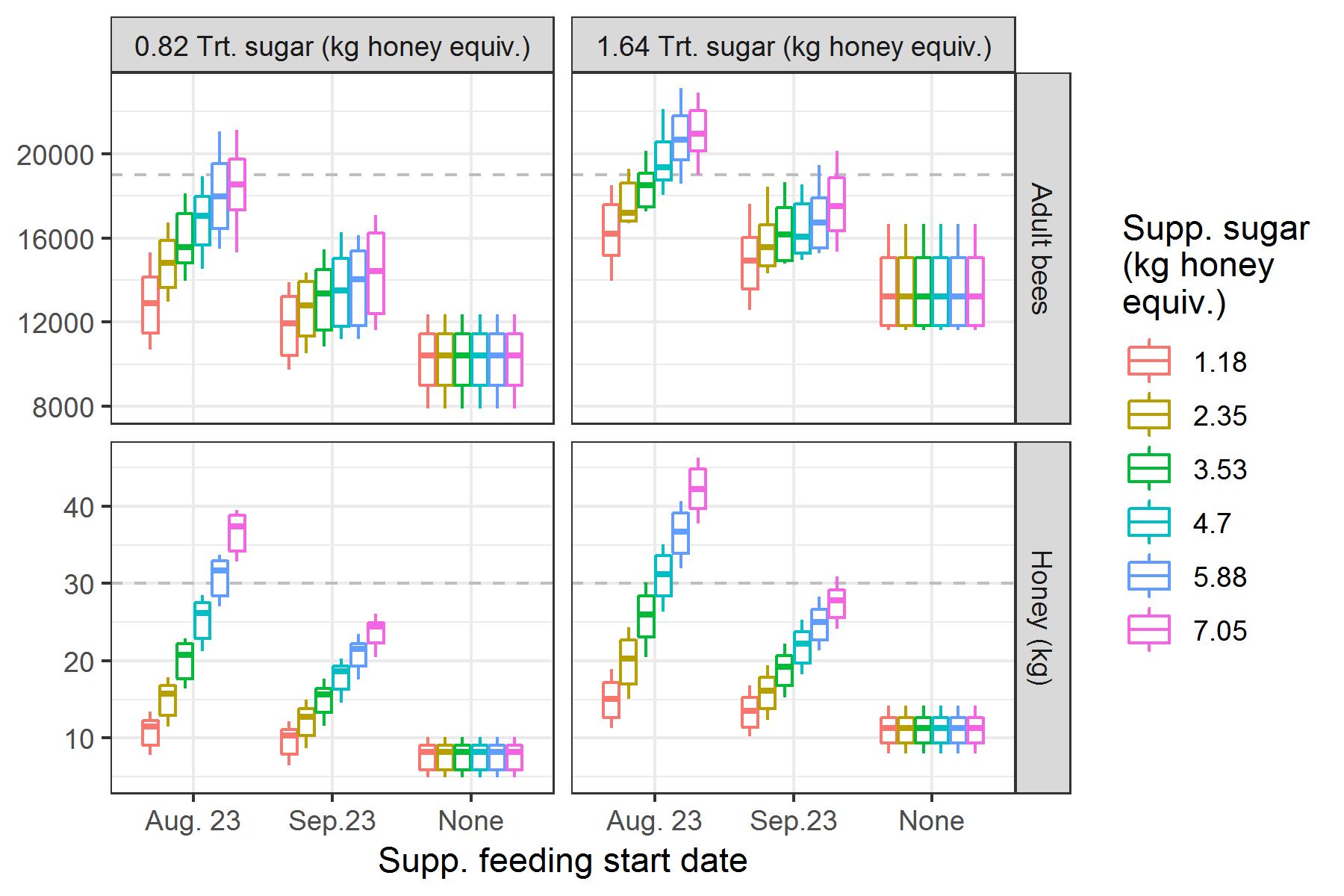


When more sugar was supplied per treatment feeding (1.64 kg honey equivalent, compare columns in Figure S11), there were more adult bees (top) and honey (bottom) on average in the fall. For supplemental feeding, more sugar per feeding produced more adults and honey by the fall (compare among colors), though the margin of benefit was greater when the feeding began earlier (compare across X axis dates). Those fed starting August 23 also received more sugar in total than those fed on September 23 due to more feedings before this October 21 analysis. When considering total amount of supplemental sugar fed, rather than amount per feeding, the earlier start date produced more fall adult bees but less fall honey (Figure S12).

Figure S12. Box plot of fall adult bees and honey stores output from the feeding-schedule BEEHAVE simulations. Plots are split as labeled by amount of sugar per treatment feeding (in kg honey equivalent) and resulting adults/honey. Along the X axis is the first date of supplemental feeding, and colors distinguish the total amount of sugar provided over the full period of supplemental feeding, limited to totals that have both August and September start dates. Dotted lines mark the overwintering survival threshold.


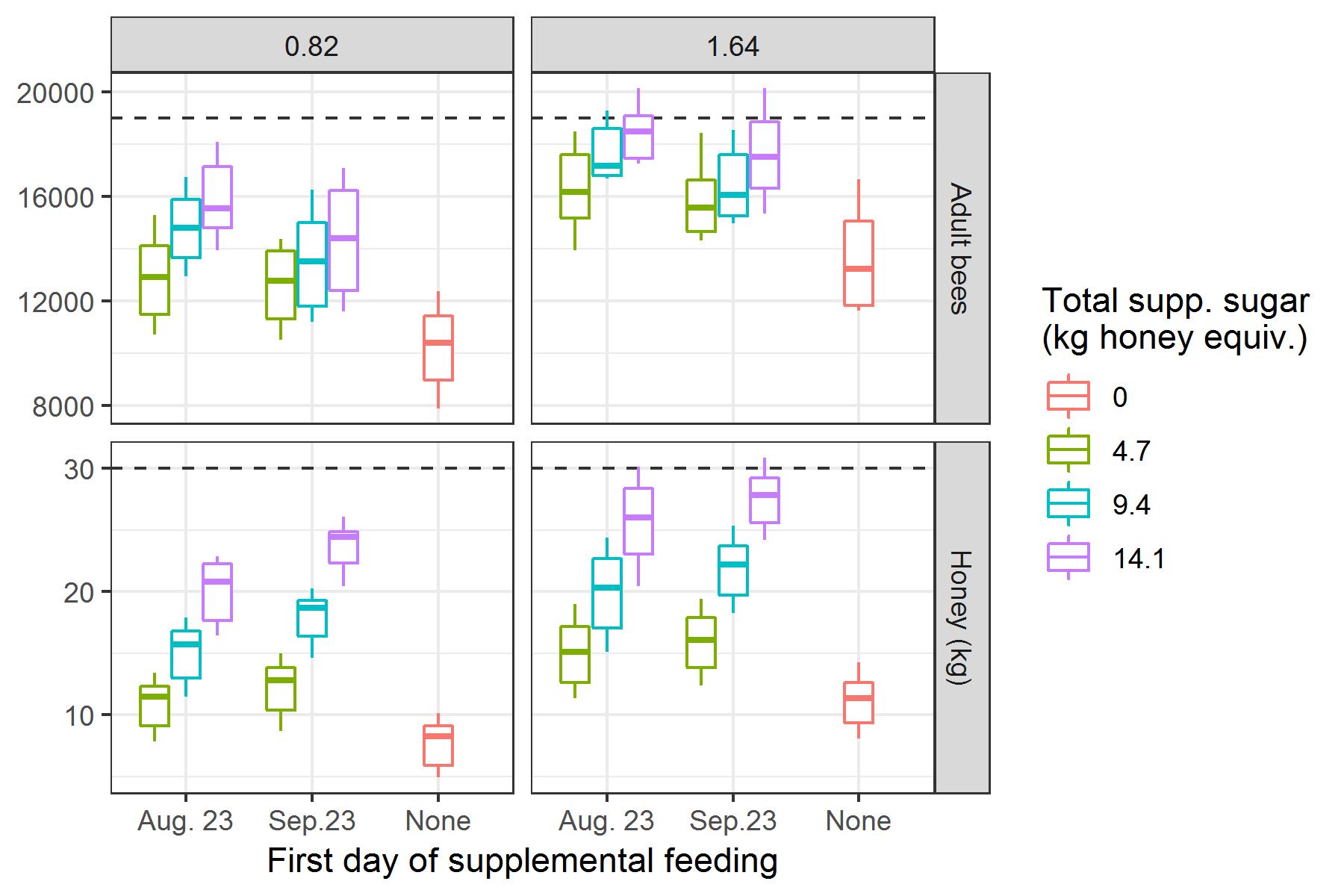


All scenarios surpassed the honey survival threshold when fed the most sugar on the earliest date. No results exceeded the survival threshold of the ratio of honey per adult bee in the fall (2.22 g/bee, not shown), though the closest value of 2.17 g/bee occurred with the high treatment and supplemental sugar amounts at the earliest supplemental feeding start date. These results are further examined and quantified in the following sections.

### Statistical analysis

Data from the feeding simulations were analyzed statistically to quantify these effects. Two statistical LME models were built, one explaining the number of adult bees in the fall and the other explaining the size of honey store in the fall. Both models were built using output values from the 288 hypothetical BEEHAVE scenarios. All values used were means of 20 replicate runs. These LME models’ outputs are presented below, then visualized and interpreted in more detail in following subsection.

#### Statistical model outputs

First, the LME model explaining the number of adult bees in the fall (Table S6) suggested that most variability across hypothetical feeding schedules was explained (R^2^_β_ = 97%) by a combination of timing and sugar amount during both treatment and supplemental feedings. Residuals appeared homoscedastic and generally normally distributed (residual plot Figure S13), meeting the assumptions of the model type.

Table S6. Results of the LME model explaining variability in the number of adult bees on October 21 (B), as output by BEEHAVE from the feeding schedule simulations. Equation form: B = Intercept + TA – SD + SA – (SD×SA).

| Random effects: |  | |  | |
| --- | --- | --- | --- | --- |
| Group | Standard deviation |  | |  |
| Apiary (8 total) | 1640 |  | |  |
| Residual | 498 |  | |  |
|  |  |  | |  |
| Fixed effects: |  | | | |
| Variable | Coefficient | Standard error | | p value |
| Intercept | 9277.31 | 595.94 | | 3.87e-07 |
| Treatment feeding amount of sugar per feeding, in kg honey equivalent (TA) | 3815.80 | 71.62 | | < 2e-16 |
| Supplemental feeding first date, with August 23 as day 0 and each additional day +1 (SD) | -33.31 | 2.69 | | < 2e-16 |
| Supplemental feeding amount of sugar fed, in kg honey equivalent (SA) | 873.02 | 23.22 | | < 2e-16 |
| SD × SA | -14.50 | 0.59 | | < 2e-16 |

Figure S13. Residual plot of the LME model of fall adult bees from the feeding-schedule simulations. BEEHAVE output is on the X axis, and LME-model output on the Y axis. Points are colored by apiary and are shown with random-effects shifts. The black diagonal line is 1:1, representing a perfect prediction. Apiaries are labeled as “CFS_[year]_[study number][apiary ID]”.


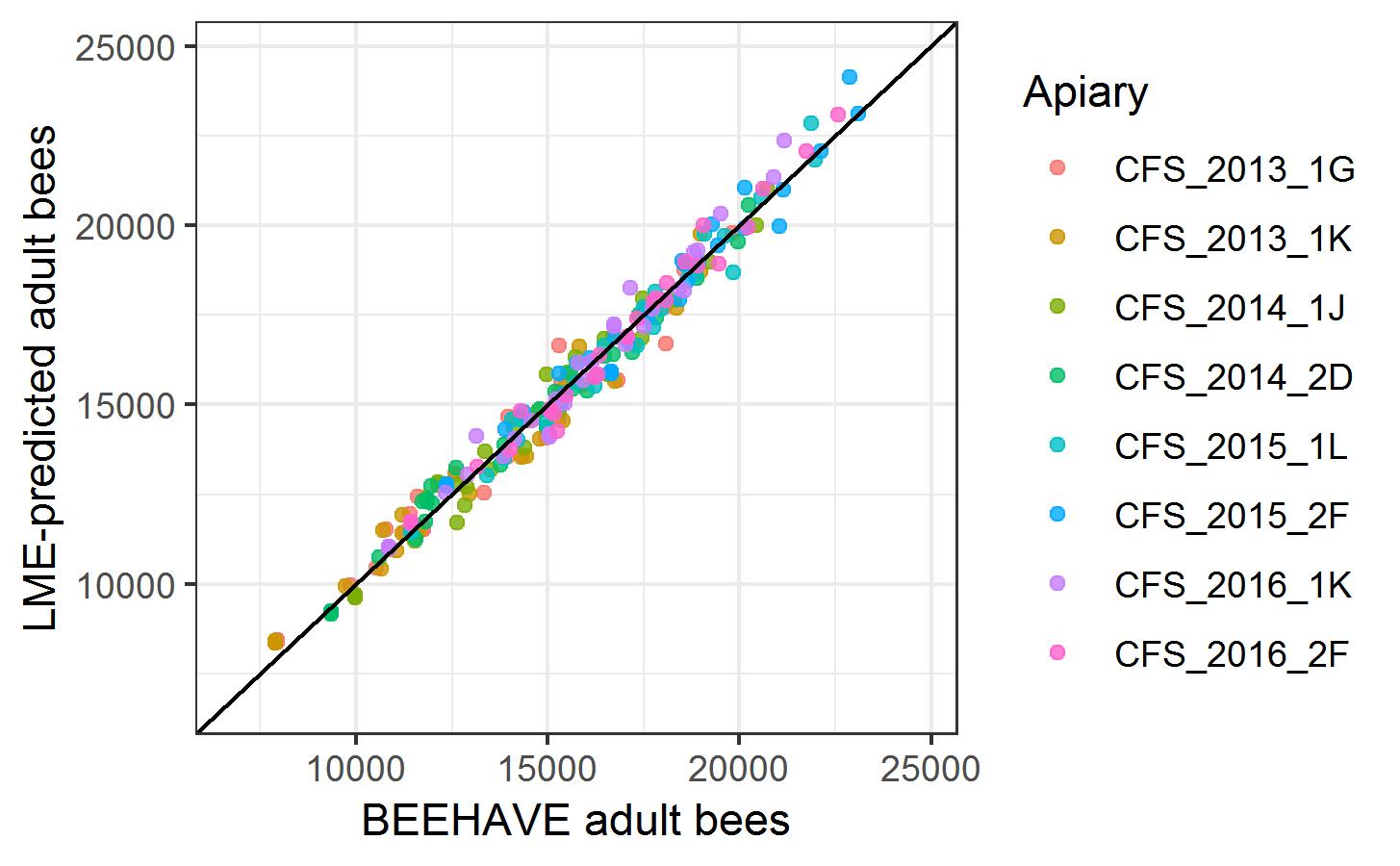


Second, the LME model explaining kg of honey in a colony in the fall was made up of the same combinations of timing and sugar amount during both treatment and supplemental feedings (Table S7). Together, all factors produced a high level of explanation (R^2^_β_ = 99%).

Table S7. Results of the LME model explaining variability in fall honey stores on October 21 (H), as output by BEEHAVE from the feeding schedule simulations. Equation form: H = Intercept + TA + SD + SA – (SD×SA).

| Random effects: |  | |  | |
| --- | --- | --- | --- | --- |
| Group | Standard deviation |  | |  |
| Apiary (8 total) | 2.518 |  | |  |
| Residual | 0.924 |  | |  |
|  |  |  | |  |
| Fixed effects: |  | | | |
| Variable | Coefficient | Standard error | | p value |
| Intercept | 0.8339 | 0.9263 | | 0.394 |
| Treatment feeding amount of sugar per feeding, in kg honey equivalent (TA) | 5.1174 | 0.1328 | | < 2e-16 |
| Supplemental feeding first date, with August 23 as day 0 and each additional day +1 (SD) | 0.03732 | 0.00499 | | 9.97e-13 |
| Supplemental feeding amount of sugar fed, in kg honey equivalent (SA) | 4.5989 | 0.04306 | | < 2e-16 |
| SD × SA | -0.07433 | 0.00109 | | < 2e-16 |

Residuals appear homoscedastic (Figure S14), but were bimodal. Stratified residual histograms showed that this was due to the apiaries having different responses to the two treatment feeding amounts (Figure S15). When treatment feedings were 0.82 kg honey equivalent, for example, this LME model systematically over-predicted fall honey in some apiaries and under-predicted it in others. This characteristic suggests that an aspect that differs among apiaries, other than feeding schedules, is not represented in this LME model. Specifically, this omitted aspect affected how the BEEHAVE-modeled bee population changed with the amount of sugar they were provided during treatment feedings. This aspect was later identified and addressed in the set of simulations targeting both study design characteristics.

Figure S14. Residual plot of the LME model of fall honey stores from the feeding-schedule simulations. BEEHAVE output is on the X axis, and LME-model output on the Y axis. Points are colored by apiary, and are shown with random-effects shifts. The black diagonal line is 1:1, representing a perfect prediction. Apiaries are labeled as “CFS_[year]_[study number][apiary ID]”.


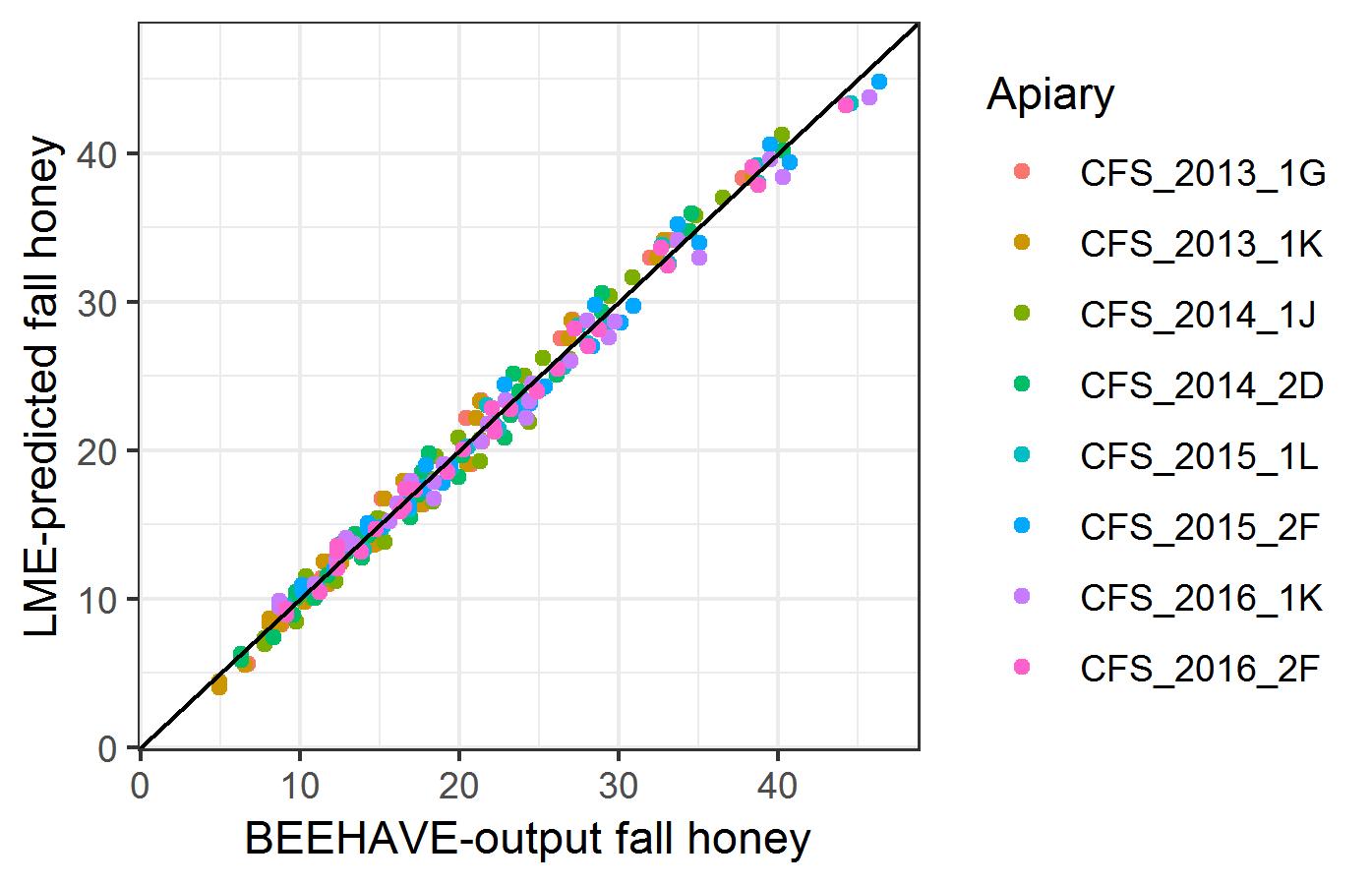


Figure S15. Histograms of residuals from the honey LME model of the feeding-schedule simulations, split by apiary and colored by amount of sugar per treatment feeding. The dotted line marks 0, a perfect prediction. Note that some apiaries have different residual directions for the same treatment sugar amount, indicating that some other apiary difference is yet unaccounted for in this model. Apiaries are labeled as “CFS_[year]_[study number][apiary ID]”.


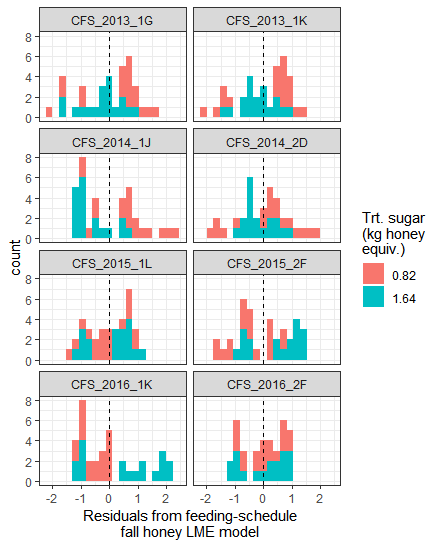


These models’ equations are next used to quantify and interpret variables’ average increases/decreases to adult bee and honey amounts on October 21 in the subsection below. Though the values are calculated as precise numbers, they are averages based on the BEEHAVE model’s outputs; colonies in reality have considerable variability in outcomes. These precise values are provided here to allow for relative comparison of impact among variables: some changes in timing or sugar amount have more impact than others.

#### Interpretation of statistical results

Using the LME equation for adult bees, the mean effects of the different feeding aspects were graphed in Figure S16, in which random-effects intercept shifts by apiary are not included. First, providing more sugar during treatment (left plot vs. right plot) improved fall colony condition. There were 3,129 more adult bees and 4.2 more kg honey on average at the greater amount of treatment sugar (1.64 kg honey equivalent, 1 L of 1:1 sugar solution). These values were true regardless of supplemental feeding details.

Figure S16. Results of the feeding-schedule LME model explaining variability of adult bee numbers in fall, graphed as mean values across different treatment sugar amounts per feeding (left and right plots), supplemental feeding start dates (colors), and supplemental sugar amounts per feeding (X axis).


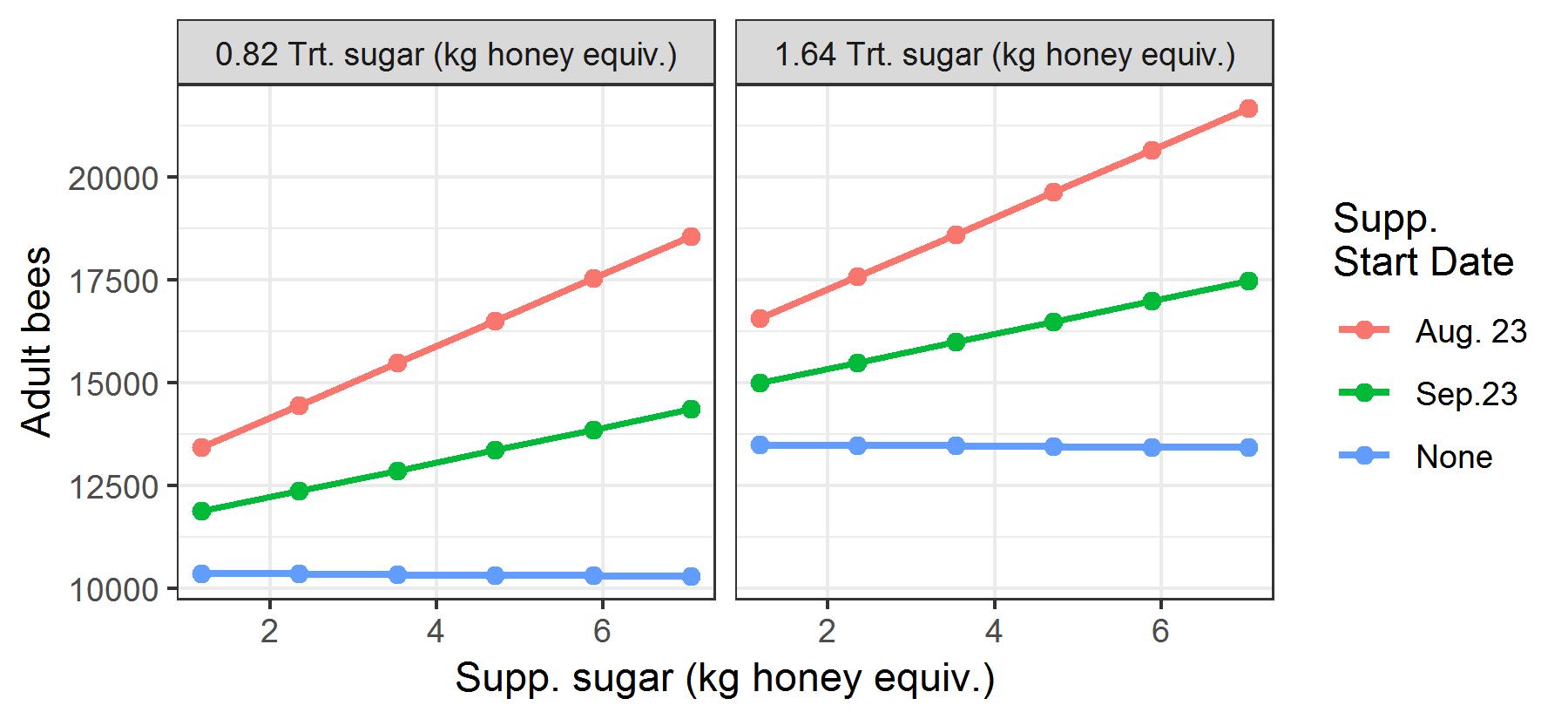


Earlier supplemental feedings resulted in more adult bees and honey on average on October 21, but the magnitude of increase (slope) depended on the amount of sugar fed. This is an interactive effect. Each additional day that supplemental feeding was delayed, there were 50 fewer adult bees and 0.04 kg less honey on average in October, calculated at the lowest simulated sugar level of 1.18 kg honey equivalent (leftmost points on both plots in Figure S16). When the sugar solution was the greatest (7.05 kg, rightmost points), the value was instead 136 fewer adults and 0.49 kg less honey per day of delay. The blue line represents no supplemental sugar fed prior to October 21, for comparison.

More sugar in supplemental feedings resulted in more adult bees and more honey on October 21 on average. At the later supplemental feeding date of September 23 (green line), each additional kg of honey equivalent provided in sugar resulted in 434 more adults and 2.6 kg more honey. At the earlier feeding date of August 23 (orange line), each additional kg of honey equivalent resulted in 873 more adults and 5.1 kg more honey.

The best-case supplemental feeding scenario, which started on the earlier date and had the most sugar, resulted in 8,200 more adult bees and 30.1 kg more honey on average compared to no supplemental feeding before October 21. These values were true regardless of treatment feeding details.

# Simulations of initial conditions

## Methods: Simulations of initial conditions

First, the distributions of initial conditions across all colonies in all studies were defined, from which low, middle, and high values were identified and systematically varied to produce a set of hypothetical initial conditions that were simulated in BEEHAVE. Second, the model outputs in the fall were graphed and statistically analyzed to link the effects of different starting conditions to fall conditions. This section details these steps.

### Setup of initial condition simulations

Once honey bee colonies were placed in their study apiaries, a Colony Condition Assessment (CCA) was conducted between June 20 and July 6 of the study year, recording each colony’s number of adults, pupae, larvae, eggs, pollen cells, and honey cells. These initial values varied by colony as well as on average by study (Figure S2 E). The values from all colonies across all studies were first combined into distributions, one per metric (e.g., all adult bee counts were pooled). Larvae and eggs in each colony were summed as uncapped brood. Honey stores in kg were derived from cell counts; for pollen, stores were translated to weight in g. The values corresponding to the 5th, 50th (median), and 95th percentiles of each distribution were calculated, and all combinations of the five metrics’ three values were generated.

However, not all combinations are relevant or realistic to consider. Scatter plots of all combinations of these five metrics were generated, and these 5th, 50th, and 95th values overlaid on each as blue squares (Figure S17). The combinations that fell in a blank area were not run in these simulations (empty blue squares). For example, 95th-percentile adult bees did not co-occur with 5th-percentile pupae numbers within individual colonies. Five of these combinations were omitted due to lack of relevance, and to reduce the number of simulations.

Figure S17. Correlation matrix of initial amounts of adult bees, pupae, brood, honey, and pollen from all colonies in all LSCFSs (black circles). The blue squares mark the 5th, 50th, and 95th percentiles of each as used in the BEEHAVE simulations; open squares were combinations not observed in the LSCFSs, therefore omitted from the simulations.


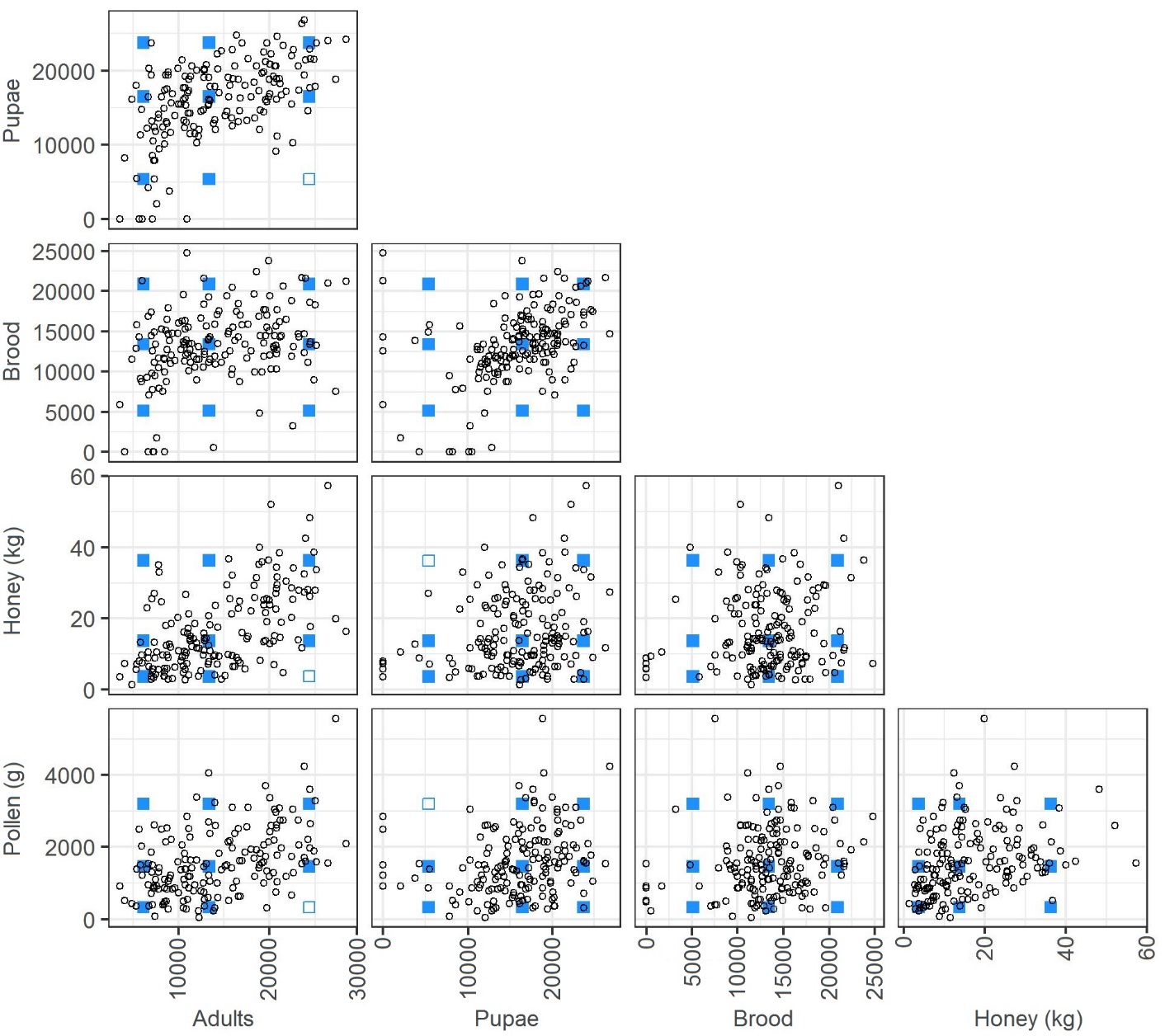


All combinations of these initial conditions were applied to the same eight apiaries used in the feeding-schedule simulations. All other unique aspects of these apiaries that varied in BEEHAVE – their weather, surrounding landscape, and feeding schedules – were used as-is, such that the only modification was their assigned hypothetical initial condition scenario.

The combinations of the low, median, and high values per metric, omitting the five unrealistic combinations, produced 156 hypothetical initial conditions. Each was applied to eight apiaries, producing 1,248 scenarios. Each scenario was repeated 20 times to incorporate stochasticity, totaling 24,960 BEEHAVE runs. The number of adult bees and size of honey store on October 21 was pulled from each of these runs, and the means of each set of 20 replicates were calculated, representing fall colony condition. These values served as the basis of the statistical analysis.

### Data analysis of initial condition simulations

The goal of this statistical analysis was to identify whether the different initial conditions of the simulated colonies impacted resulting numbers of adult bees and honey stores in the fall, and if so, to quantify those impacts. In turn, these results could be used to inform the choice of colonies to use in studies, potentially reducing overwintering losses in future studies. The scenarios that produced colonies that survived the fall in all 20 replicates (90%) were analyzed separately from the scenarios that produced some or all replicates that had died by the fall (10%), due to substantial difference in resulting values.

The main analysis of the hypothetical initial condition BEEHAVE simulations used data from the scenarios that produced surviving fall colonies in all replicates (>0 adult bees on October 21). Trends were graphed, and LME models were built for mean fall adult bees and honey using the same methodology as for the feeding schedule scenarios. In this set of simulations, five independent (or fixed) variables and their interactions were tested: the initial number of adult bees, pupae, and brood (as larvae + eggs), as well as the initial kg of honey and g of pollen in the colonies.

The hypothetical initial condition scenarios that produced at least one replicate outcome with zero fall adults were examined in a separate binary analysis. These scenarios were systematically binned into apiaries as well as the 5th, 50th, and 95th percentile values of each metric (adult bees, pupae, brood, honey, and pollen) to identify levels and combinations that produced fall colony deaths. These combinations had the highest risk of producing fall losses.

## Results: Simulations of initial conditions

Ranges of adult bees, pupae, brood, honey stores, and pollen stores of control colonies at initiation of the LSCFSs were systematically combined into hypothetical initial condition scenarios and run with BEEHAVE. Results of the effects of these scenarios’ different initial conditions are examined graphically and statistically.

### Graphical comparison

All scenarios’ results were grouped by their applied initial numbers of adult bees and colored by their initial honey store sizes in Figure S18, in which all applied apiaries and initial values of pupae, brood, and pollen are included. The overwintering survival and loss thresholds identified from the LSCFS data are shown as dotted lines for comparison. When simulated colonies began with more honey (along X axis), there were more adult bees (left plot) and honey (right plot) on average in the fall. The only colony losses occurred when colonies began with the 5^th^ percentile of honey (3.6 kg). In cases of colony death prior to October 21, honey stores from the simulations may be high because no consumption occurred after colony death. Thus, honey stores in combinations with cases of colony deaths are not indicative of colony success. More initial adult bees (compare among colors) often resulted in more fall bees and honey stores, but the effect size was considerably smaller than for initial honey stores.

Figure S18. Box plot of fall adult bees and honey stores from the initial-condition BEEHAVE simulations. Plots are split as labeled by October adult bees or honey on the Y axis. Along the X axis is the initial honey amount, and colors distinguish the initial number of adult bees. Dotted lines mark overwintering survival and loss thresholds.


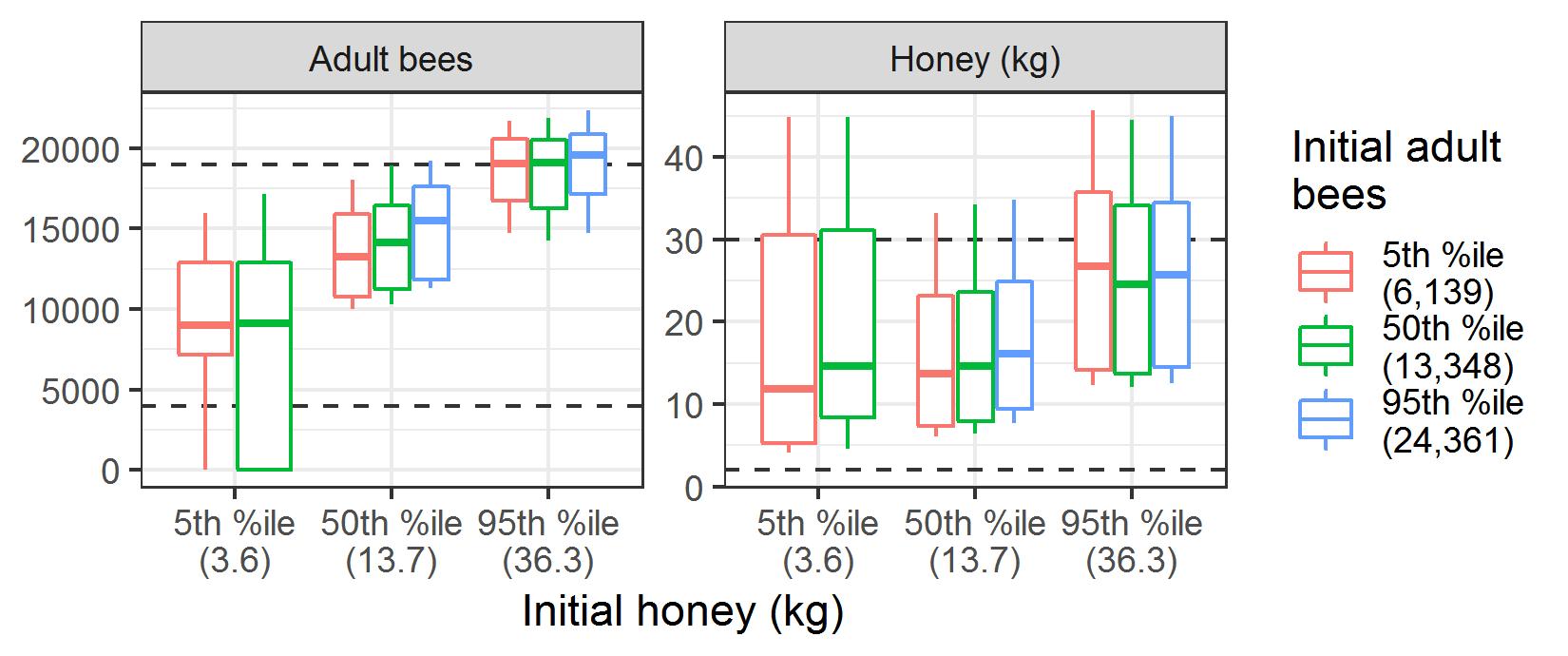


Similarly, all scenarios’ results were grouped by their applied initial pollen amount and colored by their number of pupae in Figure S19, in which all applied apiaries and initial values of honey, adult bees, and brood are included. More initial pupae (colors) meant more fall adults and honey, but initial pollen values (along the X axis) did not demonstrate a clear trend. All initial brood numbers almost completely overlapped across the full range of fall adults and honey, and therefore did not demonstrate a trend (not shown). These results are further examined and quantified by a statistical analysis.

Figure S19. Box plot of fall adult bees and honey stores output from the initial-condition BEEHAVE simulations, focusing on initial pollen and pupae. Plots are split as labeled by October adult bees or honey on the Y axis. Along the X axis is the initial pollen amount, and colors distinguish the initial number of pupae. Dotted lines mark overwintering survival and loss thresholds.


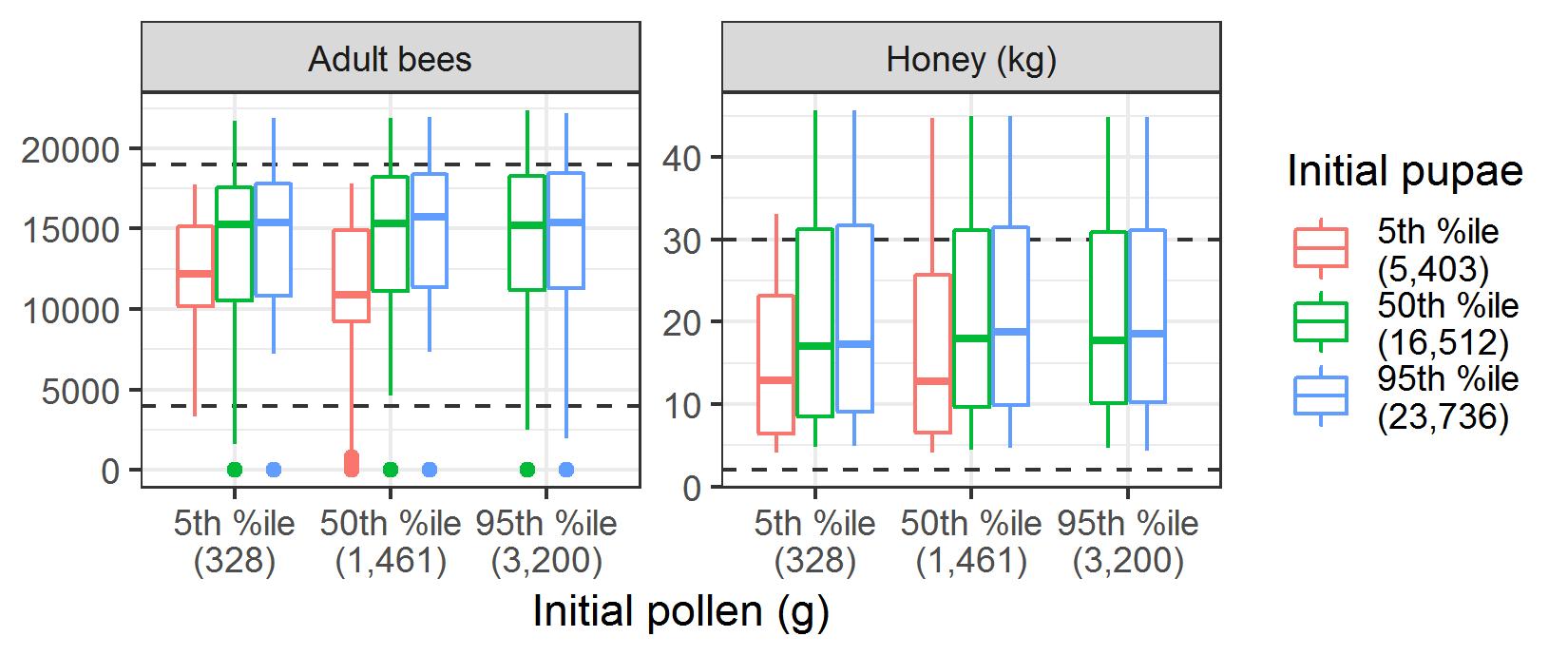


### Statistical analysis

Data from the initial condition simulations were analyzed statistically to quantify these effects. Two statistical LME models were built, one explaining the number of adult bees in the fall and the other explaining the size of honey store in the fall. All values used were means of 20 replicate runs. These LME models’ outputs are presented below, then visualized and interpreted in more detail in following subsections. Both models were built using output values from the 1,122 hypothetical BEEHAVE scenarios that had all replicates surviving to October 21, of 1,248 scenarios total. The remaining 126 scenarios were examined separately.

#### Statistical model outputs

First, the LME model explaining the number of adult bees in the fall (Table S8) suggested that most variability across hypothetical initial condition scenarios was explained (R^2^_β_ = 95%) by a combination of initial amount of honey, pollen, numbers of adult bees, and pupae. Initial brood amount made a very small impact, insignificantly improving the R^2^_β_ (R-squared difference test, Jaeger 2017). Residuals appeared homoscedastic and normally distributed (residual plot in Figure S20).

Table S8. Results of the LME model explaining variability in the number of adult bees on October 21 (B), as output by BEEHAVE from the initial condition simulations. Equation form: B = Intercept + IB + IH + IP – IO – (IB×IH).

| Random effects: |  | |  | |
| --- | --- | --- | --- | --- |
| Group | Standard deviation |  | |  |
| Apiary (8 total) | 2770 |  | |  |
| Residual | 616 |  | |  |
|  |  |  | |  |
| Fixed effects: |  | | | |
| Variable | Coefficient | Standard error | | p value |
| Intercept | 9356.252 | 982.8884 | | 2.71E-05 |
| Initial number of adult bees in colony, in thousands (IB) | 113.2386 | 5.97263 | | < 2e-16 |
| Initial amount of honey store in colony, in kg (IH) | 234.905 | 3.12745 | | < 2e-16 |
| Initial number of pupae in colony, in thousands (IP) | 35.65521 | 3.13338 | | < 2e-16 |
| Initial amount of pollen in colony, in g (IO) | -0.07954 | 0.01715 | | 3.96E-06 |
| IB × IH | -2.57508 | 0.23287 | | < 2e-16 |

Figure S20. Residual plot of the LME model of fall adult bees from the initial-condition simulations. BEEHAVE output is on the X axis, and LME-model output on the Y axis. Points are colored by apiary, and are shown with random-effects shifts. The black diagonal line is 1:1, representing a perfect prediction. Apiaries are labeled as “CFS_[year]_[study number][apiary ID]”.


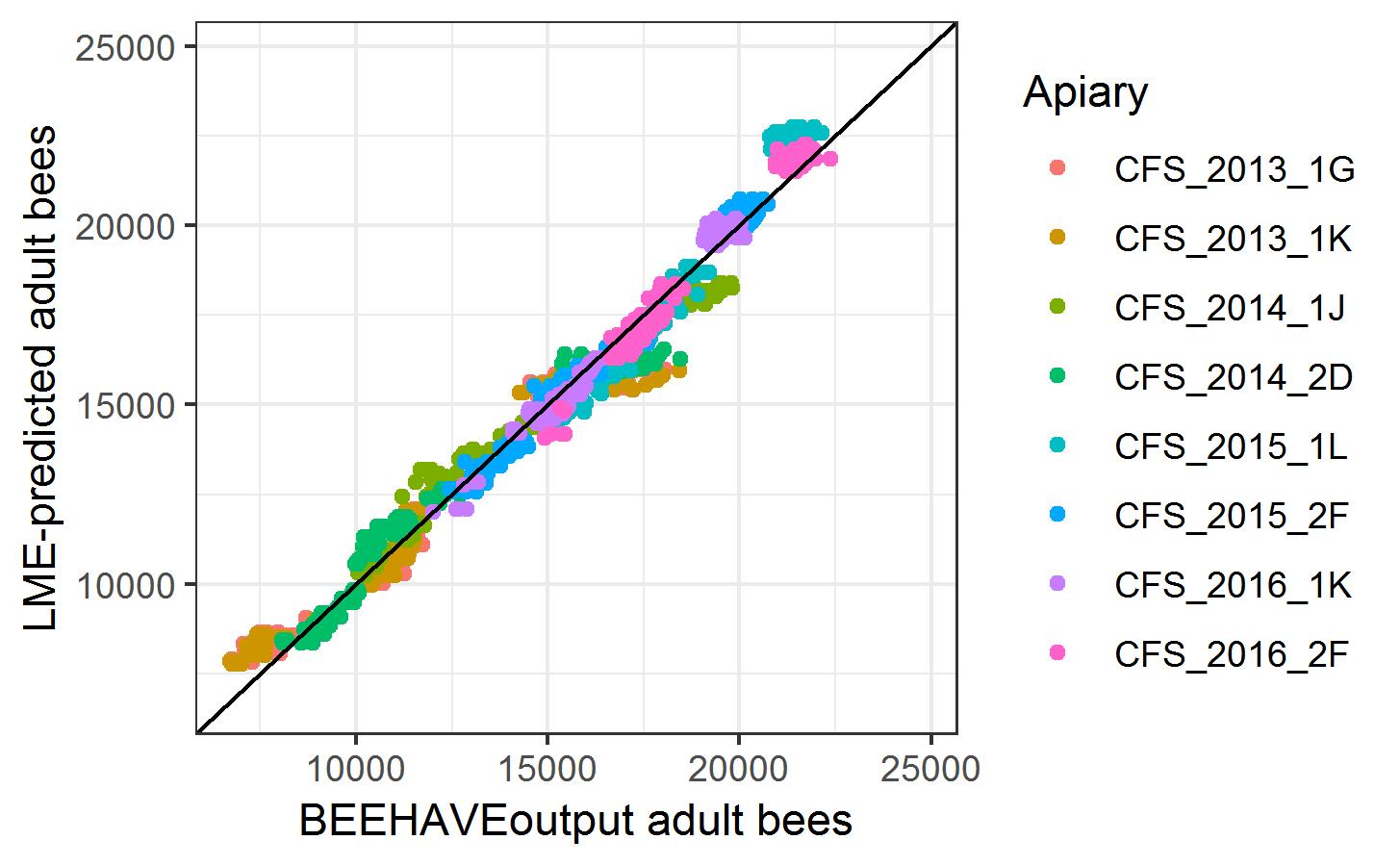


Second, the LME model explaining kg of honey in a colony in the fall (Table S9) had the same structure as the adult-bee LME model for initial conditions. Initial brood was insignificant. Together, all factors produced a high level of explanation (R^2^_β_ = 96%).

Table S9. Results of the LME model explaining variability in fall honey stores on October 21 (H), as output by BEEHAVE from the initial condition simulations. Equation form: H = Intercept + IB + IH + IP – IO – (IB×IH).

| Random effects: |  | |  | |
| --- | --- | --- | --- | --- |
| Group | Standard deviation |  | |  |
| Apiary (8 total) | 10.543 |  | |  |
| Residual | 1.537 |  | |  |
|  |  |  | |  |
| Fixed effects: |  | | | |
| Variable | Coefficient | Standard error | | p value |
| Intercept | 10.73 | 3.733 | | 0.0237 |
| Initial number of adult bees in colony, in thousands (IB) | 0.0920 | 0.0149 | | 9.25E-10 |
| Initial amount of honey store in colony, in kg (IH) | 0.3965 | 0.007801 | | < 2e-16 |
| Initial number of pupae in colony, in thousands (IP) | 0.05451 | 0.007816 | | 5.25E-12 |
| Initial amount of pollen in colony, in g (IO) | -0.000177 | 4.28E-05 | | 3.96E-05 |
| IB × IH | -0.003251 | 0.000581 | | 2.76E-08 |

Residuals appear homoscedastic (Figure S21), but their distribution was non-normal. Stratified residual histograms revealed that the three levels of initial honey, and the apiaries within them, had different directionalities of response (Figure S22. For example, when the initial honey amount was set at the 95^th^ percentile, some apiaries’ fall honey amounts were systematically under-predicted, while those of other apiaries were over-predicted. This characteristic suggests that there is a missing apiary-specific aspect that modifies the effect of initial honey amount on fall honey stores. This aspect is identified and incorporated into the fall honey LME model of the simulations targeting both study design characteristics.

Figure S21. Residual plot of the LME model of fall honey stores from the initial-condition simulations. BEEHAVE output is on the X axis, and LME-model output on the Y axis. Points are colored by apiary, and are shown with random-effects shifts. The black diagonal line is 1:1, representing a perfect prediction. Apiaries are labeled as “CFS_[year]_[study number][apiary ID]”.


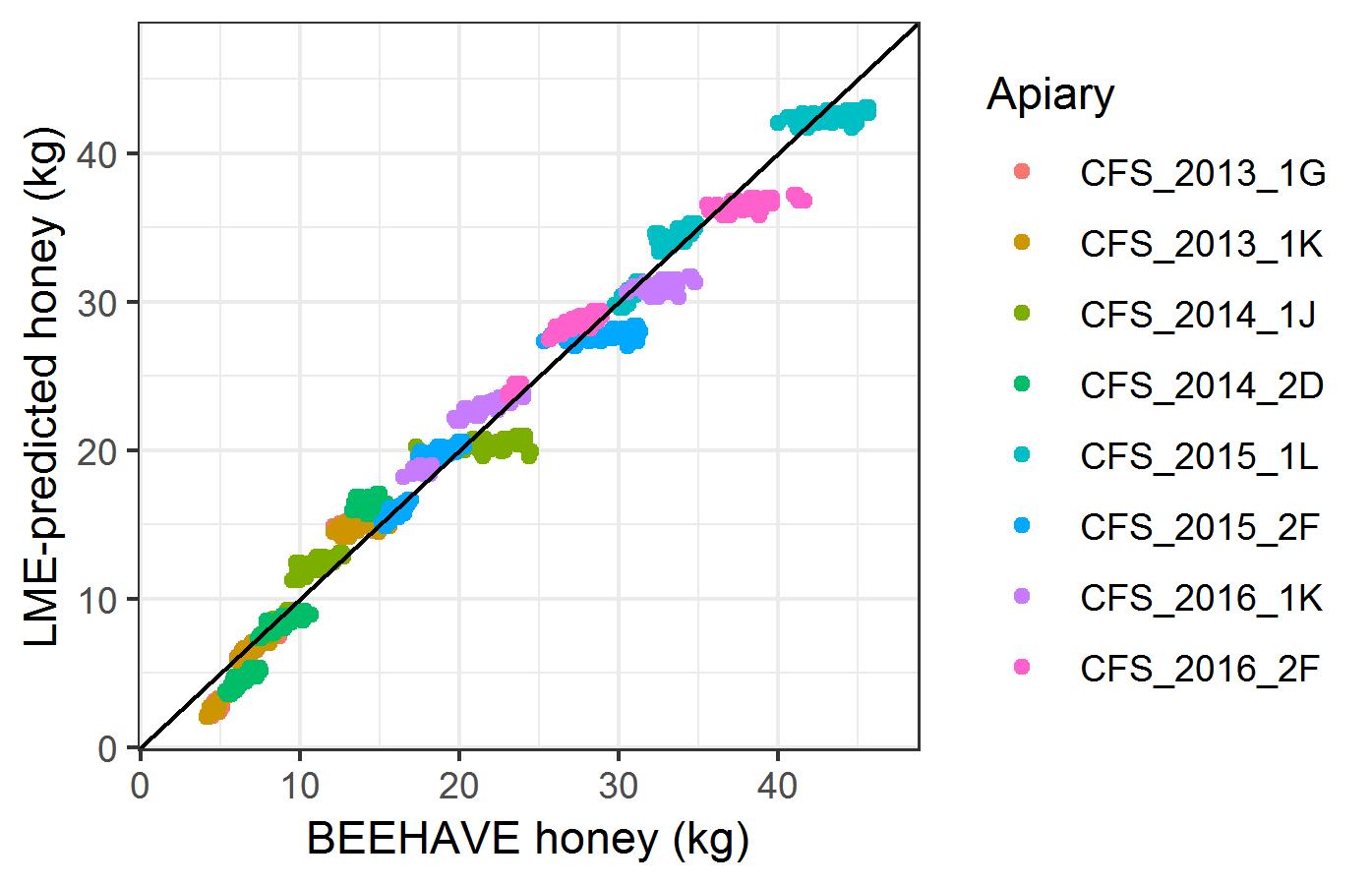


Figure S22. Histograms of residuals from the honey LME model of the initial-condition simulations, split by apiary and colored by amount of initial honey. The dotted line marks 0, a perfect prediction. Note that different apiaries have different residual directions for the same initial honey amount, indicating that some other apiary difference is yet unaccounted for in this model. Apiaries are labeled as “CFS_[year]_[study number][apiary ID]”.


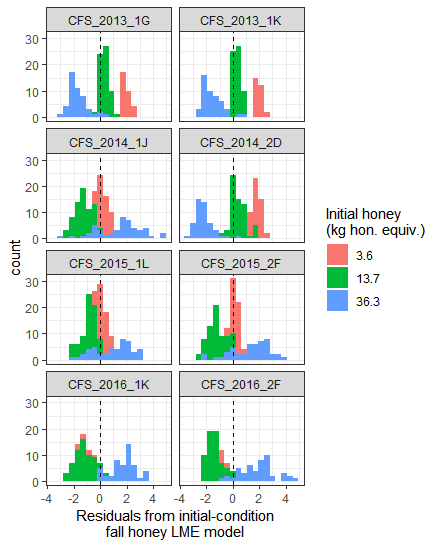


These models’ equations are used to quantify and interpret average increases/decreases in adult bee and honey amounts on October 21 in the following subsection. The values are not intended to serve as precise predictions, but to provide relative comparison of impact among variables: some initial conditions have more impact than others.

#### Interpretation of statistical results

The factor with the largest influence on colony condition simulated in the fall was the size of honey stores at study initiation. This effect is illustrated in relation to fall adult bees in Figure S23, limited to the median initial pupae and pollen values, as an example. The simulated range of initial honey amounts spans the X axis. Incrementally, at the 5th percentile of initial adult bees (orange line), each additional kg of initial honey store resulted in 201 additional adult bees and 0.4 kg more honey in the fall on average. At the 95^th^ percentile of initial adults (blue line), each additional kg of initial honey only led to 172 more fall bees and 0.3 kg more honey, demonstrating the interactive effect between initial honey and adult bees.

Figure S23. Average effects of initial honey and adult bees, from the initial-condition LME model explaining variability of adult bee numbers in fall. Points are graphed as mean values across different initial honey amounts (X axis) and initial adult bee numbers (colors). This is an example set at the median initial pollen and pupae values.


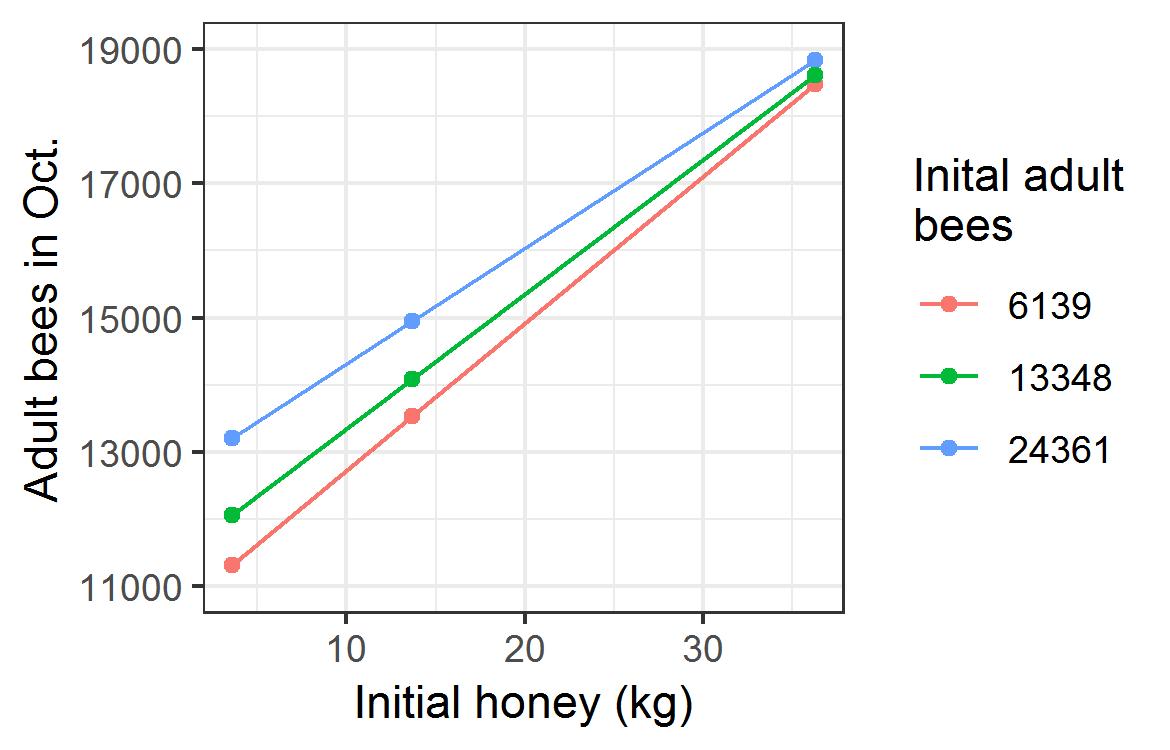


Similarly, at the 5^th^ percentile of initial honey (leftmost points), each additional 1000 initial adult bees resulted in 104 additional fall adult bees and 0.08 kg more fall honey on average (when the colony survived early in the season). At the 95^th^ percentile of initial honey (rightmost points), each additional 1000 adults led to only 20 more fall adult bees on average, but reversed to decrease the amount of fall honey by 0.03 kg likely due to more bees’ consumption. Having more initial adults was of more benefit in simulated colonies with less initial honey. Across the 90-percentile ranges of initial honey and adult values (5^th^ to 95^th^ percentiles of both), the average number of fall bees increased by 7,525 and the average honey store increased by 11.8 kg.

Initial pupae had relatively less influence on fall adult bee numbers. Figure S24 displays the same adult bee range as Figure S23, and is limited to the median initial honey and adult bee values, as an example. For each additional 1000 initial pupae in a simulated colony, there were 36 more fall adult bees and 0.05 kg more honey on average. This translates to a 654-bee and 1.0-kg increase across the 90-percentile range of pupae. Across the 90-percentile range of pollen, the number of fall adult bees only decreased by 228 and the amount of honey by 0.5 kg, a negligible change compared to the ranges produced in this model, 8,407 fall adult bees and 13.8 kg fall honey.

Figure S24. Average effects of initial pupae and pollen, from the initial-condition LME model explaining variability of adult bee numbers in fall. Points are graphed as mean values across different initial pupae numbers (X axis) and initial pollen amounts (colors). This is an example set at the median initial adult bee and honey store values.


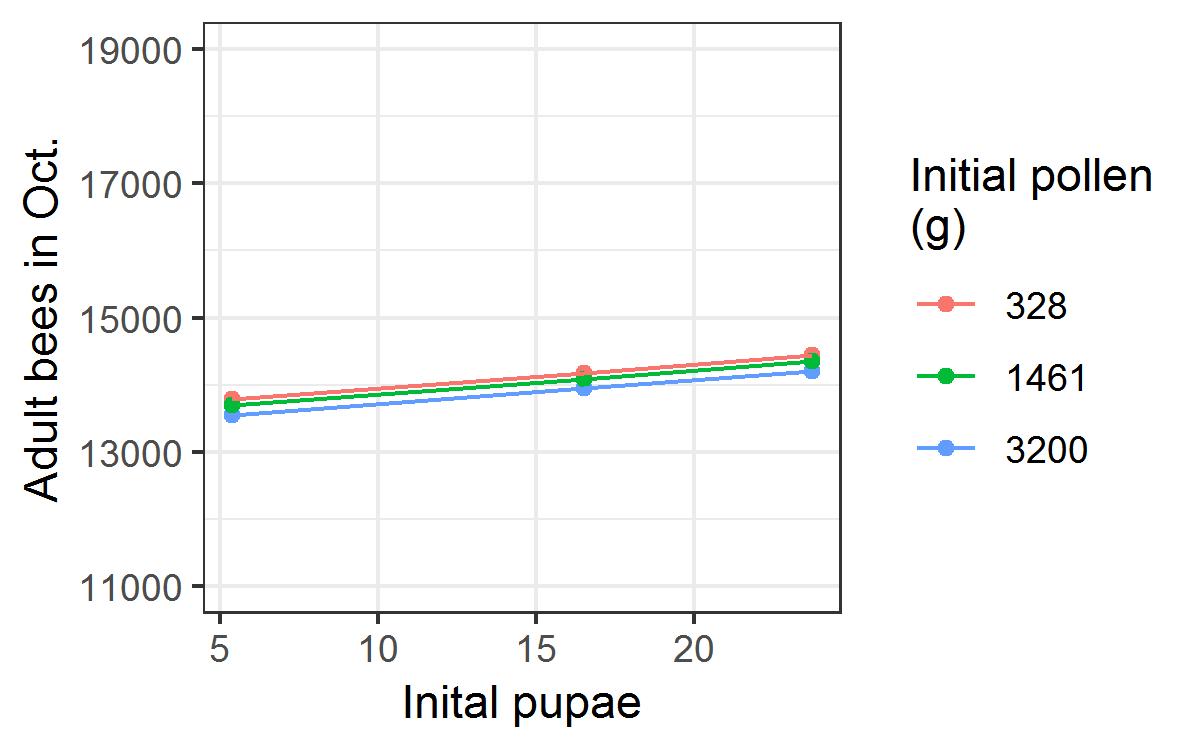


### Binary analysis of fall losses from initial conditions

Among all hypothetical initial condition scenarios run through BEEHAVE, 10% (126/1,248) produced replicates that died before October 21. All occurred when the initial amount of honey was set to the 5th percentile value, suggesting that colonies with too little initial honey may not survive to the fall.

Among the scenarios at this low honey level, all were assigned either the 5th or 50th percentile of initial adult bees; however, the 95th percentile of initial adults and 5th percentile of initial honey was not run because there was no record of this combination among the seven LSCFSs examined (Table S10). The median level of initial adults produced more losses than the low level of adults, which suggests that more consumers early in the season could strain low honey resources to the point of colony death in the simulations.

Table S10. Number of hypothetical initial condition scenarios that resulted in some or all replicate colony deaths prior to October 21. These counts are limited to the scenarios with 5th percentile initial honey amount only (3.6 kg), since no losses occurred at the higher amounts. NA indicates that the combination was not run, since 5th-percentile initial honey and 95th-percentile initial adults did not co-occur in LSCFSs.

|  | Initial adult bees | | |
| --- | --- | --- | --- |
| Apiary | 5^th^ percentile (6,139) | 50^th^ percentile (13,348) | 95^th^ percentile (24,361) |
| CFS_2013_1G | 0 | 17 | NA |
| CFS_2013_1K | 0 | 19 | NA |
| CFS_2014_1J | 0 | 0 | NA |
| CFS_2014_2D | 0 | 4 | NA |
| CFS_2015_1L | 0 | 2 | NA |
| CFS_2015_2F | 0 | 2 | NA |
| CFS_2016_1K | 20 | 21 | NA |
| CFS_2016_2F | 20 | 21 | NA |

Additionally, the different apiaries had different results. The 2016 apiaries had the most losses, followed by 2013, with 2014 and 2015 producing the fewest losses. Similarity within years suggests that weather may be an impactful factor in distinguishing apiary response to fall survival.

# Simulations targeting study design characteristics: Graphical comparison of results

From these targeted BEEHAVE simulations, adult bee numbers and honey stores on October 21 were grouped and graphed by feeding schedule characteristics in Figure S25, in which all apiaries and initial conditions are included. Comparing between the left and right plots, more sugar supplied during treatment feedings led to more adult bees and honey stores in the fall. The total amount of sugar fed during supplemental feeding is represented by colors, showing that more sugar led to more adults and honey in the fall. At the highest level and at the later feeding start date, there is some tapering of the effect in adults. The two supplemental feeding start dates on the X axis allow for a comparison of an earlier feeding schedule with less sugar per feeding against a later feeding schedule with more sugar per feeding. Though the same amounts of sugar are ultimately provided, starting earlier appears to produce more fall adults, but less fall honey.

Figure S25. Box plot of fall adult bees and honey stores output from the targeted BEEHAVE simulations, focusing on feeding schedule aspects. Plots are split as labeled by amount of sugar per treatment feeding, and resulting adults/honey. Along the X axis is the first date of supplemental feeding, and colors distinguish the total amount of sugar provided from the start date through October 21. Dotted lines mark overwintering survival and loss thresholds.


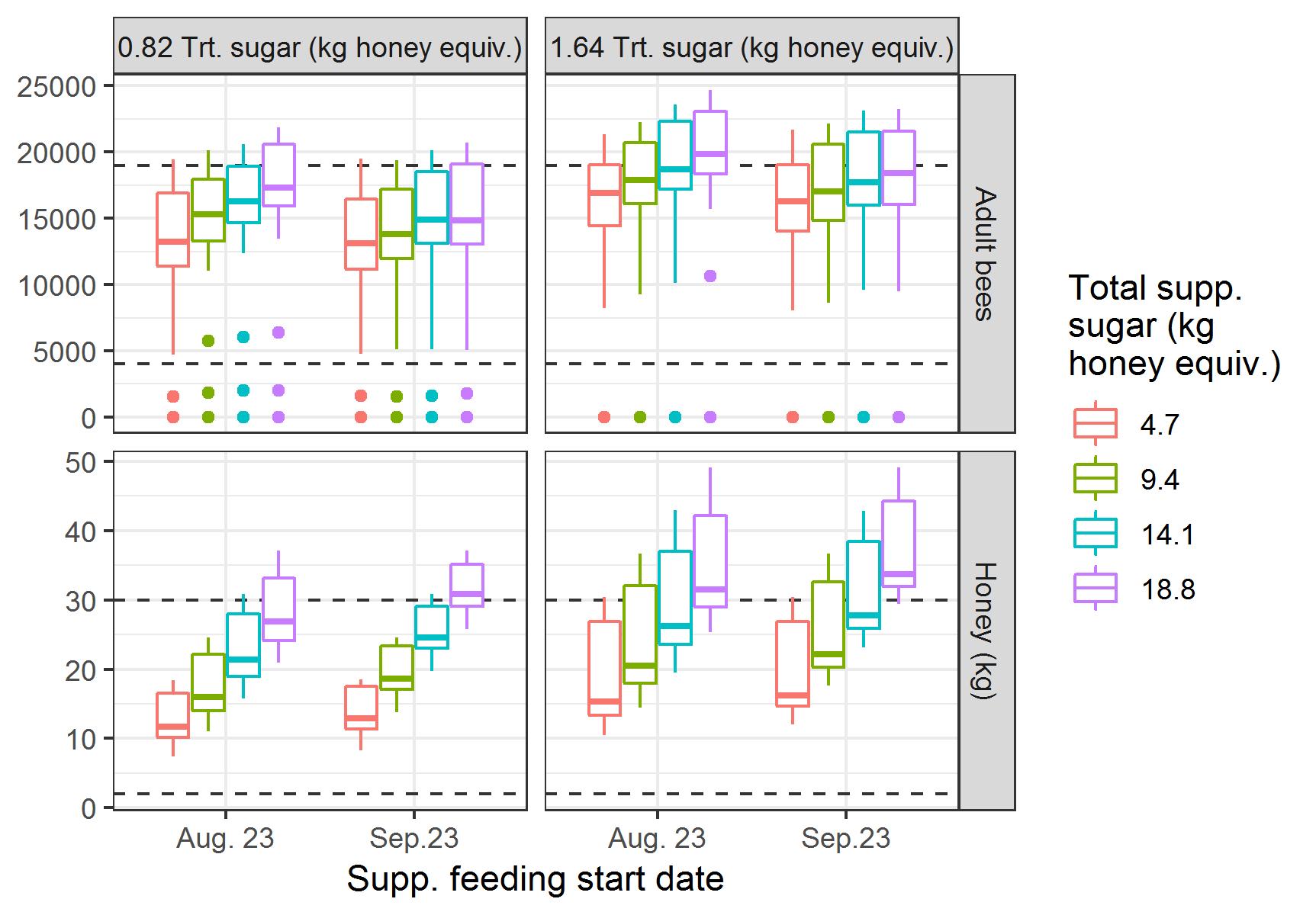


In Figure S26, the X axis and coloring instead reflect initial colony conditions; all apiaries and supplemental feeding schedules are included. Conditions that produced colonies that died by the fall (in some or all replicates) were limited to the lowest initial honey level; at that level, colonies with more initial adult bees resulted in more fall deaths. This combination of scenarios also produced more fall honey, since more was uneaten. As above, more sugar per treatment feeding meant more fall adults and honey. However, the jump is noticeably higher especially for fall honey at the combination of the highest amount of initial honey and the most treatment feeding. More initial honey consistently produced more fall adults and honey, but more initial adult bees had mixed results. At the median level of initial honey, more initial adults increased fall metrics, but the outcome from number of initial adults varied at the other levels.

Figure S26. Box plot of fall adult bees and honey stores output from the targeted BEEHAVE simulations, focusing on initial condition aspects. Plots are split as labeled by amount of sugar per treatment feeding, and resulting adults/honey. Along the X axis is the initial honey amount, and colors distinguish the initial number of adult bees. Dotted lines mark overwintering survival and loss thresholds.


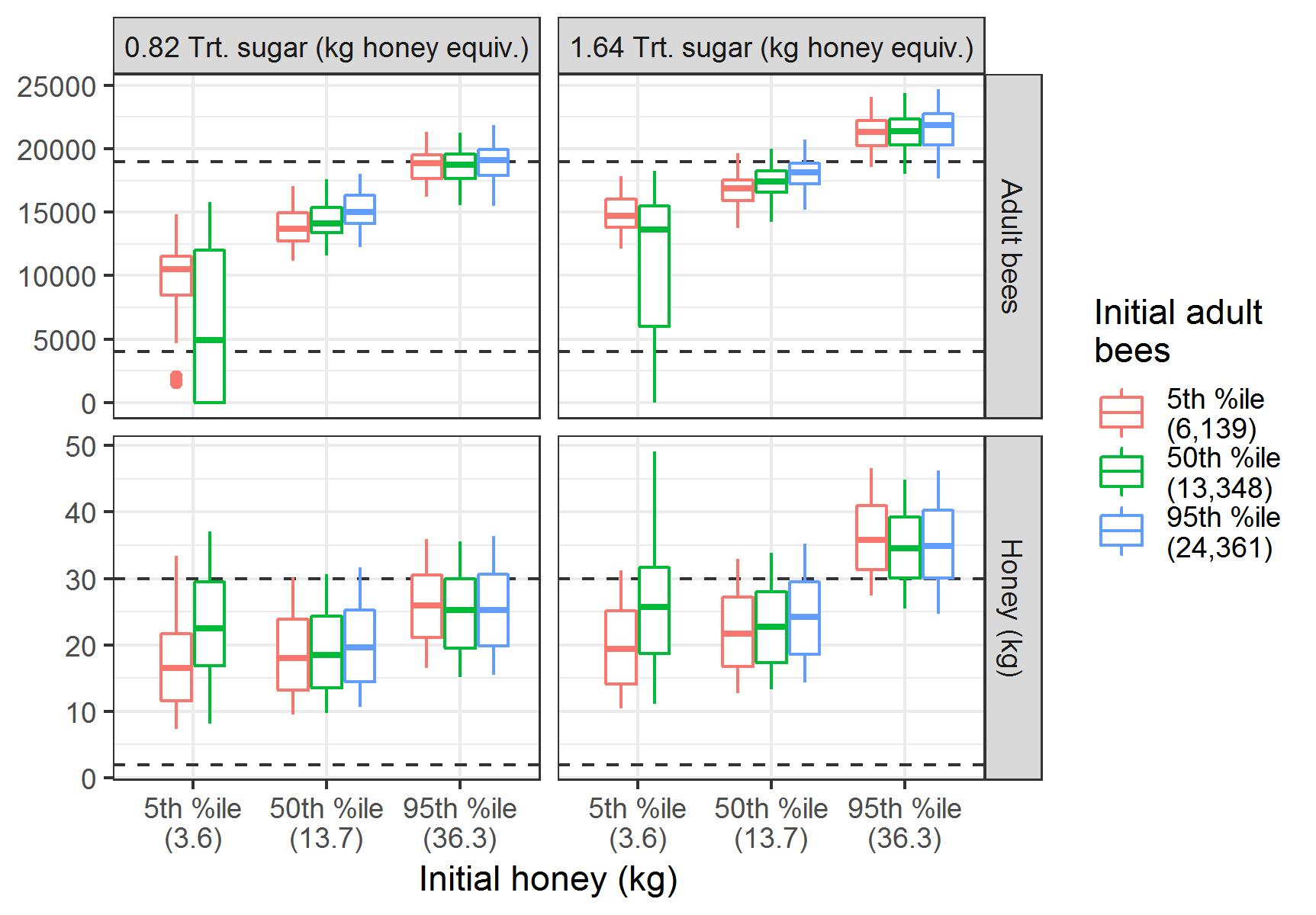


# Comparison to relationships in LSCFS data

## Methods: Comparison to relationships in LSCFS data

The systematic BEEHAVE simulations and statistical analyses suggested that a set of study design aspects (initial colony condition and feeding patterns) had a meaningful impact on fall colony conditions. We carried out an additional check to assess whether these findings were qualitatively discernable in the original LSCFS data, as measured. Patterns were not expected to be clear-cut because the studies and colonies within them varied in multiple ways, but relative comparisons were possible.

To do so, CCA measurements were averaged within apiaries, i.e., among the two control colonies included in each. This approach was also applied in the calibration of BEEHAVE to account for the considerable variability observed between control colonies that experienced very similar conditions throughout the study period. Colonies that died prior to the fall were not included in averages. Without using BEEHAVE data, these mean LSCFS data points were graphed alongside the impactful aspects to check for visible trends. Both metrics of fall adult bees and fall honey stores were graphed, as well as their overwintering thresholds determined from the LSCFS data and actual colony overwintering outcomes.

## Results: Comparison to relationships in LSCFS data

We can qualitatively compare the findings of the BEEHAVE simulation analysis with the measured fall outcomes of the colonies in the seven LSCFSs. Studies differed from each other in multiple aspects of study design. However, trends identified from the simulations may be apparent in the study data as well. Did colonies with early and high supplemental feeding, more treatment feeding, more initial honey, and more initial adults result in more fall honey and adult bees?

With each point reflecting a mean apiary value, Figure S27 shows the mean measured honey stores at their first colony assessment on the X axis, and mean measured fall adult bees or honey on the Y axis. General positive trends are visible, in that low initial honey values often led to low fall adults and honey, and vice versa, corroborating the simulation analysis result. Points are also colored by the supplemental feeding start date; note that studies with early dates also provided more sugar (see Figure S3), so date and amount are confounded. Apiaries with earlier and higher supplemental feeding were the only points above the fall honey survival threshold. Conversely, those fed in December and late October had less fall honey. These trends were more mixed in relation to fall adult bees, suggesting that these supplemental feeding schedules affected fall honey stores more directly than they affected fall bee populations.

Figure S27. Graphs comparing mean initial honey amount (X axis) and supplemental feeding start date (colors) of all apiaries to resulting fall adult bee numbers (left) and honey stores (right). Each point represents one apiary, such that initial honey values are the mean of two colonies. Note that earlier supplemental feedings also had more sugar per feeding. Points are shaped by the colonies’ overwinter results as labeled. Dotted lines mark overwintering thresholds.


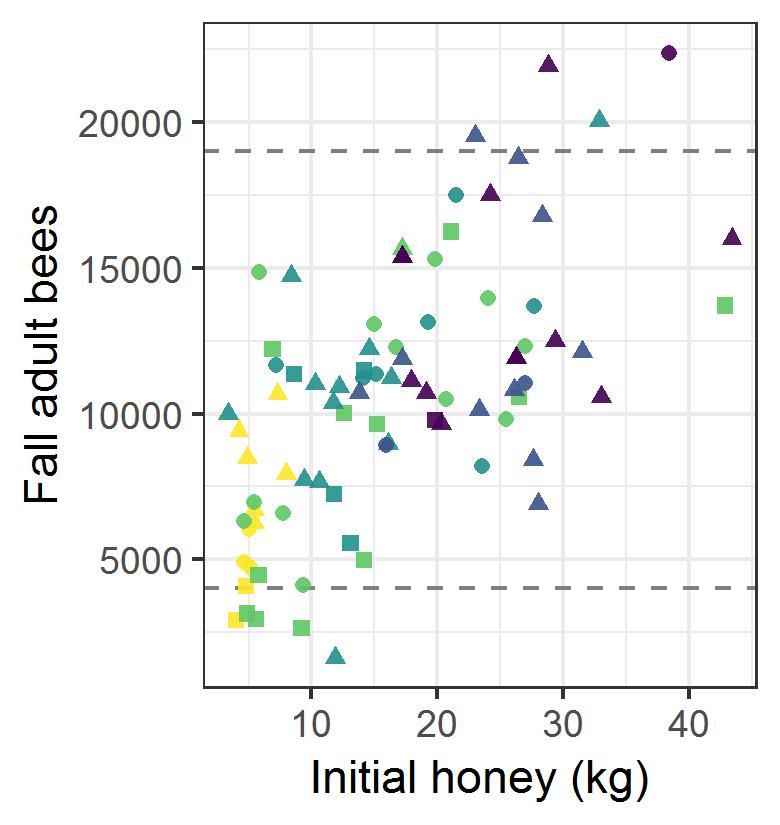

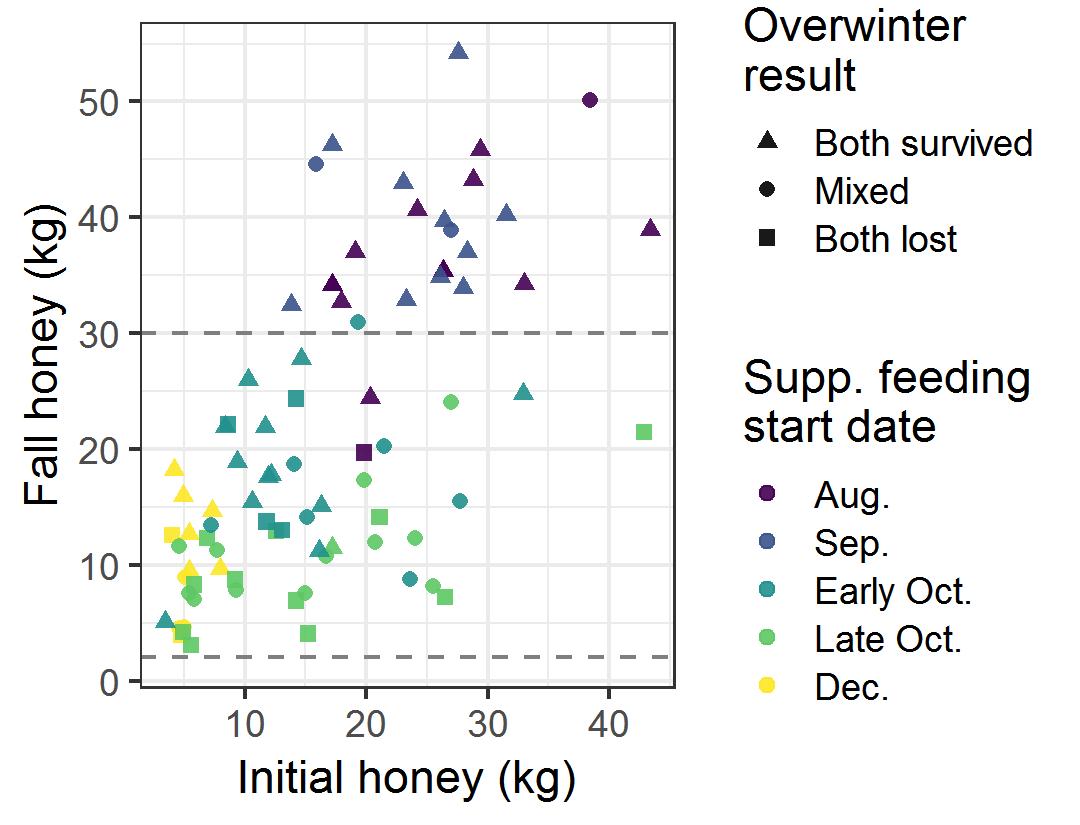


Initial adult bees and mean treatment sugar amount were similarly compared to fall colony conditions in Figure S28, but associations were less consistent. With initial adult bees on the X axis, the distribution of points is wider (lower correlation), as expected from the smaller impact of initial adult bees found in the simulations’ statistical analyses. Treatment amounts were not consistently indicative of fall honey, as this is likely dominated by the strong explanations in the previous figure. Lower treatment sugar was more often associated with fewer fall adults, but these colonies also had fewer initial adult bees, less initial honey, and late supplemental feeding. Though these variables’ associations are not discernable when presented in this context, this is expected since treatment sugar and initial adult bees are dominated and confounded by initial honey and supplemental feeding aspects. Parsing these complex relationships necessitated the systematic BEEHAVE simulations and statistical analyses, which identified individual variables’ impacts while controlling for all others.

Figure S28. Graphs comparing mean initial number of adult bees (X axis) and mean amount of sugar per treatment feeding (colors) of all apiaries to resulting fall adult bee numbers (left) and honey stores (right). Each point represents one apiary, such that initial adult bee numbers are the mean of two colonies. Points are shaped by the overwinter result as labeled. Dotted lines mark overwintering thresholds.


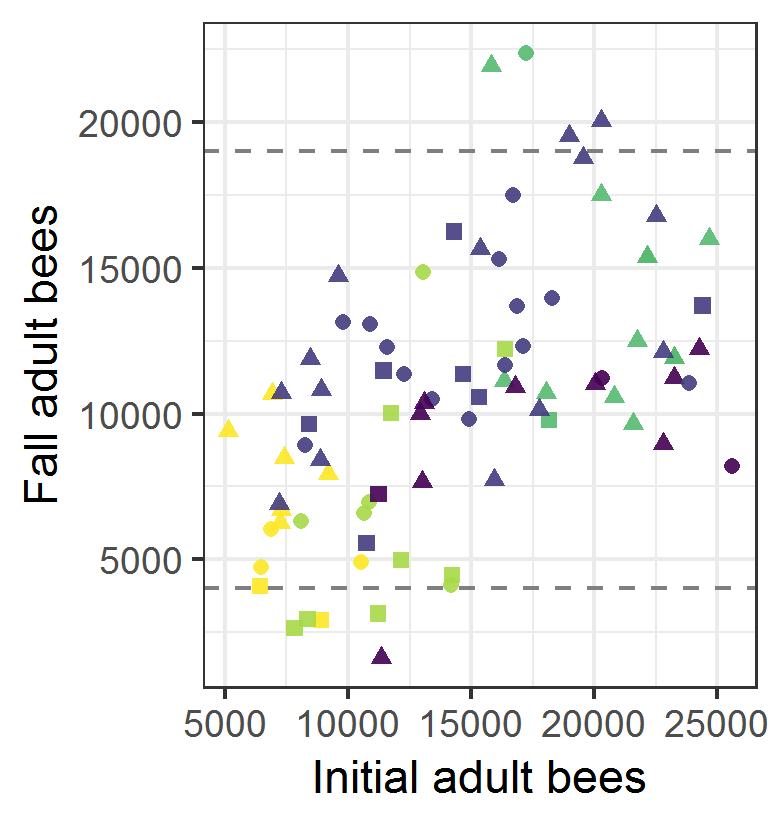

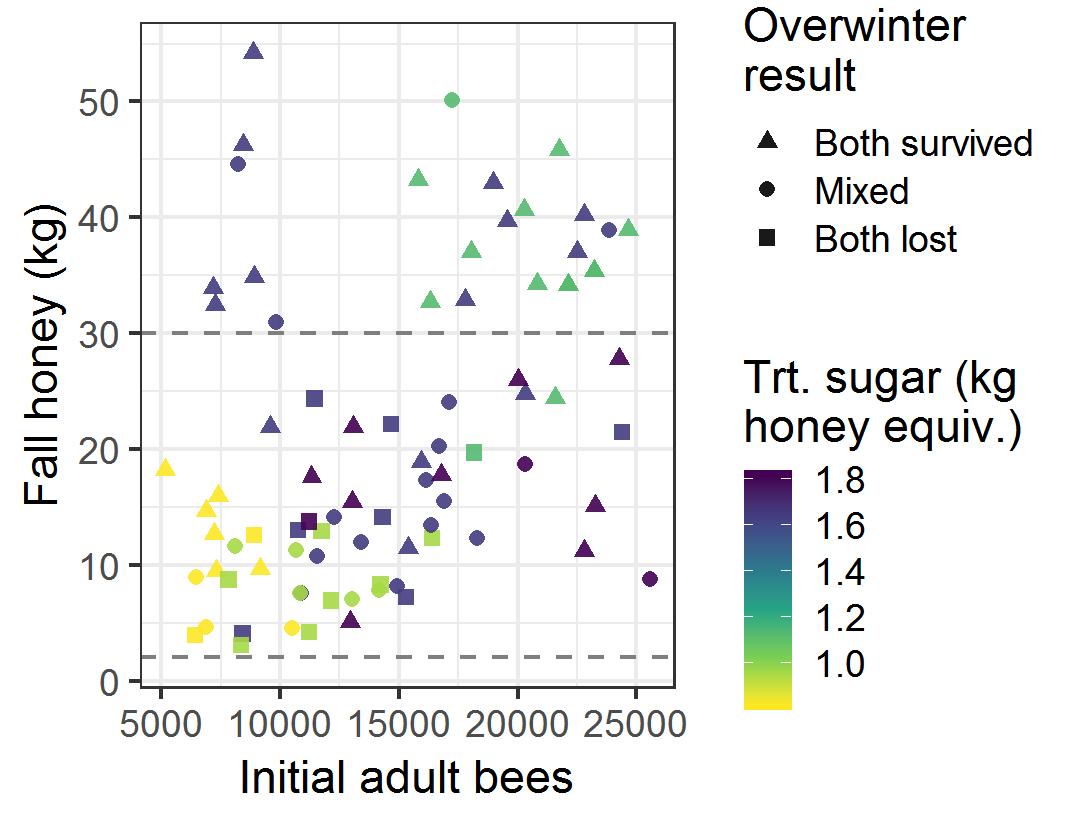

Supplement: Supplementary file 1 — Supporting information. [file ETC-39-2286-s001.docx]
